# Industrial Vestiges: Legacies of Ancillary Impacts of Resource Development

**DOI:** 10.1007/s41636-023-00389-0

**Published:** 2023-06-12

**Authors:** Anatolijs Venovcevs

**Affiliations:** UiT: The Arctic University of Norway, Institute for Archaeology, History, Religious Studies, and Theology, Brelia L103, Hansine Hansens Veg 18, 9019 Tromsø, Norway

**Keywords:** Labrador, Kola Peninsula, Russia, industrial archaeology, mining towns, 20th century

## Abstract

This article offers a different way to understand the heritage of extractive industries by exploring the material afterlives of what has been termed the “ancillary impacts of resource development”—a variety of quarries, forest cuts, transportation corridors, and power lines that surround industrial operations, especially those created in areas distant from established industrial population centers. To study this, the article expands upon the concept of “vestige” to explore the landscapes around two single-industry mining towns in Kola Peninsula, Russia, and in Labrador, Canada, by specifically focusing on two abandoned quarries located in each. The results highlight the need to explore developments that trail behind industrial settlement of colonial hinterlands. By focusing specifically on the afterlives of such developments, the article demonstrates how chronological and geographical boundaries of resource extraction are blurred over time, creating a deep, unruly, self-perpetuating set of legacies.

## Resumen

Este artículo ofrece una manera diferente de comprender el patrimonio de las industrias extractivas mediante la exploración de las vidas materiales posteriores a lo que se ha denominado los “impactos secundarios del desarrollo de recursos”: una variedad de canteras, cortes de bosques, corredores de transporte y líneas eléctricas que rodean las operaciones industriales, especialmente aquellas creadas en áreas distantes de los centros de población industrial establecidos. Para estudiar esto, el artículo amplía el concepto de “vestigio” para explorar los paisajes alrededor de dos pueblos mineros de una sola industria en la península de Kola, Rusia, y en Labrador, Canadá, centrándose específicamente en dos canteras abandonadas ubicadas en cada uno. Los resultados resaltan la necesidad de explorar los desarrollos que vienen después de los asentamientos industriales del interior colonial. Al centrarse específicamente en las vidas posteriores de tales desarrollos, el artículo demuestra cómo los límites cronológicos y geográficos de la extracción de recursos se desdibujan con el tiempo, creando un conjunto de legados profundos, rebeldes y que se perpetúan a sí mismos.

## Résumé

Cet article propose une manière différente de comprendre le patrimoine des industries d'extraction par l'étude des vies postérieures matérielles de ce qui est désigné sous le terme d'« impacts accessoires du développement de ressources »—plusieurs carrières, abattages de forêts, corridors de transport et lignes électriques entourant les exploitations industrielles, en particulier ceux créés dans des zones distantes des centres établis de population industrielle. Aux fins de cette étude, cet article expose en détail le concept de « vestige » pour explorer les paysages environnant deux villes minières à industrie unique dans la Péninsule de Kola en Russie et dans le Labrador au Canada, et s'intéresse tout particulièrement à deux carrières abandonnées dans chacune d'entre elles. Les résultats soulignent la nécessité d'une étude des développements faisant suite à l'implantation industrielle des arrières-pays coloniaux. En s'intéressant plus particulièrement aux vies postérieures de ces développements, l'article démontre comment les limites chronologiques et géographiques de l'extraction de ressources deviennent floues au cours du temps, créant de ce fait un ensemble enraciné, chaotique et se répétant sans cesse de transmissions.

## Introduction

Since the emergence of industrial archaeology in the 1970s, archaeologists have exerted a tremendous amount of work to tracing the development and evolution of extractive industries that have fundamentally transformed the human and physical geography of the planet. Similar research, in the associated field of industrial heritage, has worked to understand the afterlives of deindustrialization. While this work is extensive, one area that has remained generally underreported and undertheorized is the geographically dispersed network of supportive industries that trailed in the wake of industrial operations when they entered new and previously unindustrialized areas (though see literature such as Lawrence et al. [2016, 2017] and Baeten et al. [2018] for excellent work on the legacies of water consumption/infrastructure). As just one example of such gaps, in their seminal textbook on industrial archaeology, Marilyn Palmer and Peter Neaverson (1998:29–32) focus on adits, waste tips, shafts, power sources, and smelting mills as key features that help to identify former mining landscapes overlooking the broader secondary operations that often went into supporting these industries. This gap is present outside of archaeology as well. In a recent article by Tolvanen et al. (2019), the authors’ systematic review on the economic, social, and legal issues surrounding the contemporary mining industry in the Arctic neglects to consider the fact that mining operations carry with them ancillary impacts that have far-reaching and long-lasting consequences on the human and nonhuman environments.

This article takes a slightly different approach by performing an archaeological investigation on the ongoing legacies of what Arn Keeling (2010:235–236) has referred to as the ancillary impacts of resource extraction. Keeling, a historical geographer, used the term “ancillary impacts” to capture a broad variety of secondary effects of mining operations that stem from an influx of outside workers and creation of support industries to feed the demands of heavy industry when it enters new regions that often lie peripheral to larger industrial centers and developed support networks. In Keeling’s case study, Uranium City, Saskatchewan, those impacts included logging of old growth forests and hydroelectric developments visible up to a hundred kilometers away from the site of extraction. To name just a few other examples, ancillary impacts can include activities such as mineral survey work, new agricultural enterprises, road and railroad construction, quarrying, and an increase in hunting and fishing from a large influx of outside workers (Lee and Boutin 2006; Keeling 2010:235–236; Keeling and Sandlos 2017:386; Parlee et al. 2018). I am employing a concept from historical geography in this article to better grasp the secondary impacts of industry because the tools of geography are well positioned to understand how conceptualizations and uses of space are, and have been, transformed over time—a necessary framework when exploring the dispersion and profusion of industrial effects.

It should be noted that, while I use the term “ancillary” and “secondary,” at times interchangeably, throughout this article, these words are not intended to demean the significance of these industries or the people who worked in them. Rather, ancillary industries are positioned always in relationship to and in service of larger industrial operations. They would not exist without the main industrial operations they are tied to and often are owned or subcontracted by the main industrial actor in a given region. The “ancillary-ness” of ancillary operations may not necessarily be apparent in the densely populated and economically diversified regions of the world, but in the current case studies that deal with single industrial mining towns located deep in the boreal forest, ancillary operations are easier to trace since almost every contemporary object there exists in support of or because of the mining industry.

My fascination with this topic comes from the fact that, in the eyes of the general public, secondary industrial operations are often either overlooked and taken for granted pieces of infrastructure—for example powerlines, highway connections, and power plants—or perceived as necessary eyesores and localized environmental damage—such as gravel pits and prospecting cut-lines. Despite such perceptions, a closer look reveals that they are miniature worlds in and of themselves capable of assembling and projecting their own material agency. Though the research presented here can stand alone, I would be remiss to not qualify that much of my inspiration for this topic comes from my life and work in Newfoundland and Labrador in eastern Canada—a large, diffusely populated province with a rich assemblage of wide-spanning infrastructures, gravel pits, and other supportive industries that surround scattered single-industrial communities.

In this context, understanding the ancillary operations and their effects is important because they expand the industrial transformations of relatively concentrated extraction and processing operations up to a regional level. By focusing specifically on that which remains after ancillary operations have ceased serving their purpose, the application of archaeological methods can play a key role in shaping scholarly understanding about how regions become transformed through industrial processes and how those transformations perpetuate themselves beyond their human intentions. This understanding is particularly vital for cases where industrial development takes place in circumpolar regions. As John Sandlos and Arn Keeling (2012:8–9) have argued, Arctic and sub-Arctic territories have limited ability for redevelopment and economic diversification due to low population densities, poor agricultural potential, relative isolation, and the dependency on a few extractive staples that are vulnerable to boom-and-bust cycles. This means that after a powerful bust, abandoned objects can sit idly in perpetuity, creating a mass of unwanted, unmanaged, and unruly heritage that has an impact for years to come (Olsen and Pétursdóttir 2016; Keeling and Sandlos 2017).

Therefore, the purpose of this article is to explore this topic by suggesting one way that archaeology can contribute to the understanding of material legacies of such discontinued ancillary operations. To do so, I further develop and apply the term “vestige” to examine how resource extraction leaves indomitable marks on the landscape that cut across temporal and geographical boundaries through a variety of post-use trajectories. Ultimately, I try to address the following questions: what roles do ancillary operations of resource extraction assume after their abandonment and how can their post-abandoned states affect the ways in which we may think about the temporal and geographical limits of modern and historical resource extraction?

To do so, I focus on the vestiges of two features near two 20th-century single-industry mining towns located in similar taiga environments—a quartzite quarry of Rizh-Guba (Риж-Губа) outside of Monchegorsk, Murmansk Region, Russia, and the sand pits in Wabush, Labrador, Canada. At one point, these places served key roles in initializing and supporting resource extraction in their respective regions as part of a broader network of ancillary operations. However, they have since become abandoned through changing economic, social, and geological realities.

## Archaeology of Twentieth-Century Single-Industry Mining Towns

The research presented here straddles two archaeological subdisciplines—industrial archaeology and contemporary archaeology. As an industrial archaeology project, it focuses on the industrial developments, evolution, and remains of single-industry towns in northwestern Russia and northeastern Canada that developed around the extraction of minerals during the mid-20th century. It borrows approaches from industrial archaeology such as identifying entire landscapes of industry, processes of operation, and houses and institutional buildings of workers and managers. Unlike much of industrial archaeology that has been practiced to date (Palmer and Neaverson 1998; Casella and Symonds 2005; Martin 2012; Palmer and Orange 2016), however, its purpose has been analytical and theoretical rather than descriptive and protectionist. The mines and their associated towns in my case studies are not threatened with decay and demolition but are instead active, living, and breathing industrial communities that continue to serve their original purpose. This creates a dearth of formally recognized heritage—the stuff that constitutes my cases studies is too recent and too useful to be readily thought of as belonging to a valued past by the people who live there, at least not to a past that can be codified and protected in any meaningful way (R. Harrison 2020).

Considering this, the work borrows approaches from contemporary archaeology defined as the archaeology of the recent past and the present and posed variously as the archaeology of postmodernity, supermodernity, Capitalocene, or the Anthropocene (Buchli and Lucas 2001; R. Harrison and Schofield 2010; González-Ruibal 2019; Stewart et al. 2020). While the purpose of this research is not to refute or advance contemporary archaeology’s various definitions, archaeology of 20th-century single-industry mining towns is intrinsically rooted in the global flows of resources and capital that have characterized economic realities over the last 70 years and the exploitative “mass destruction” (LeCain 2009) mining practices that are a major contributing factor to the proposed new geological age. In particular, my research draws upon the ideas of Bjørnar Olsen and Þóra Pétursdóttir in tracing the legacies of the Anthropocene to consider all pasts—wanted and unwanted, recognized and unrecognized—as part of one collective and involuntary unruly heritage that is becoming a greater and greater norm in a world dominated by unmanageable materiality (Olsen and Pétursdóttir 2016; Olsen and Vinogradova 2019). The removal of distinction between valued and unvalued material pasts provides a useful analytical framework to archaeologically approach my case studies regardless of local perceptions and heritage designations.

The dual subdisciplinary pillars of industrial and contemporary archaeology allow for concepts to blend or reflect off each other to produce deeper insights into both. For example, the definition of contemporary archaeology advanced by Rodney Harrison and John Schofield (2010:1) as the study of “late-modern, post-industrial societies” falters in the industrial hinterlands of Russia and Canada where life is dominated by single-industry employers in a way not dissimilar to the early and mid-20th century, or for that matter, the company towns of the preceding two centuries. These places are not postindustrial, nor can it be said that they are post- or late-modern when they are materially entrapped in the mid-20th-century systems of life and industrial production (Venovcevs 2021). Meanwhile, while Palmer and Orange (2016:85–86) advance aerospace, automotive, and cellular telecommunications industries as topics for a future industrial archaeology, my research underscores the fact that more traditional industrial archaeology topics such as mines and mining towns have not gone away. If anything, the mineral extraction industry has gained an even greater preeminence in the recent decades as the exponential global growth in consumption and the aforementioned developments of new technologies have led to exponential growth in size and scale of resource extraction and processing. For example, the nickel and copper smelting plant of Severonickel associated with my case study in Monchegorsk, stands to become one of the largest processing plants of its type in the world—in large part due to the demands for nonferrous metals as part of the so-called green shift (Nilsen 2021).

## Monchegorsk and Rizh-Guba

**Fig. 1:**
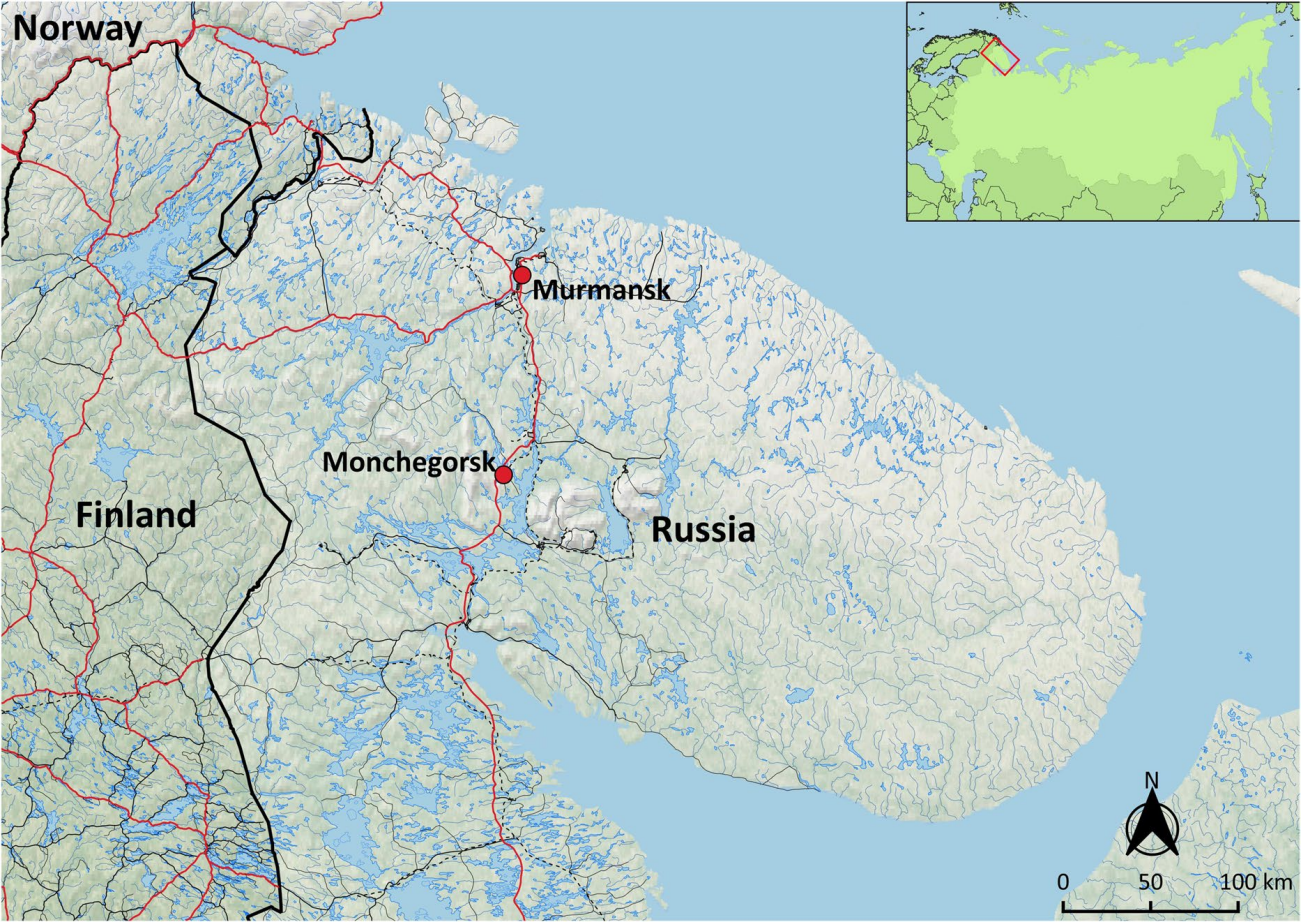
Town of Monchegorsk in the Murmansk Region, northwest Russia. (Map by author, 2020.)

It is this case study in Monchegorsk that I turn to first, located on the Kola Peninsula in the northwest corner of Russia (Fig. 1). Before rapid industrialization in the 20th century, the Indigenous Sámi occupied the region for millennia. While gradual contact with larger European powers introduced new technology, beliefs, and economic systems, much of their traditional way of life remained relatively unaffected into the early 20th century (Gutsol et al. 2007:9–25; Wheelersburg and Gutsol 2008).

While the Russian revolution brought about some early changes in the social and economic life of the Sámi, the most radical changes started a decade later in the 1930s when the Soviet Union began a massive redevelopment campaign to transform its northern and eastern regions into vast industrial areas that transformed largely Indigenous home regions to industrial peripheries servicing distant population centers within a planned economy. Mining developments along with the associated impacts such as road and railroad construction, hydroelectric projects, and establishment of military bases marginalized, resettled, and outright destroyed Indigenous settlements and traditional territories (Gutsol et al. 2007:26–47; Allemann 2013). For example, the Russian geologist who discovered the ore deposits in Monchegorsk proclaimed with pride how a town replaced the homes of the Sámi who once lived there (Lukichev 1993:48).

**Fig. 2:**
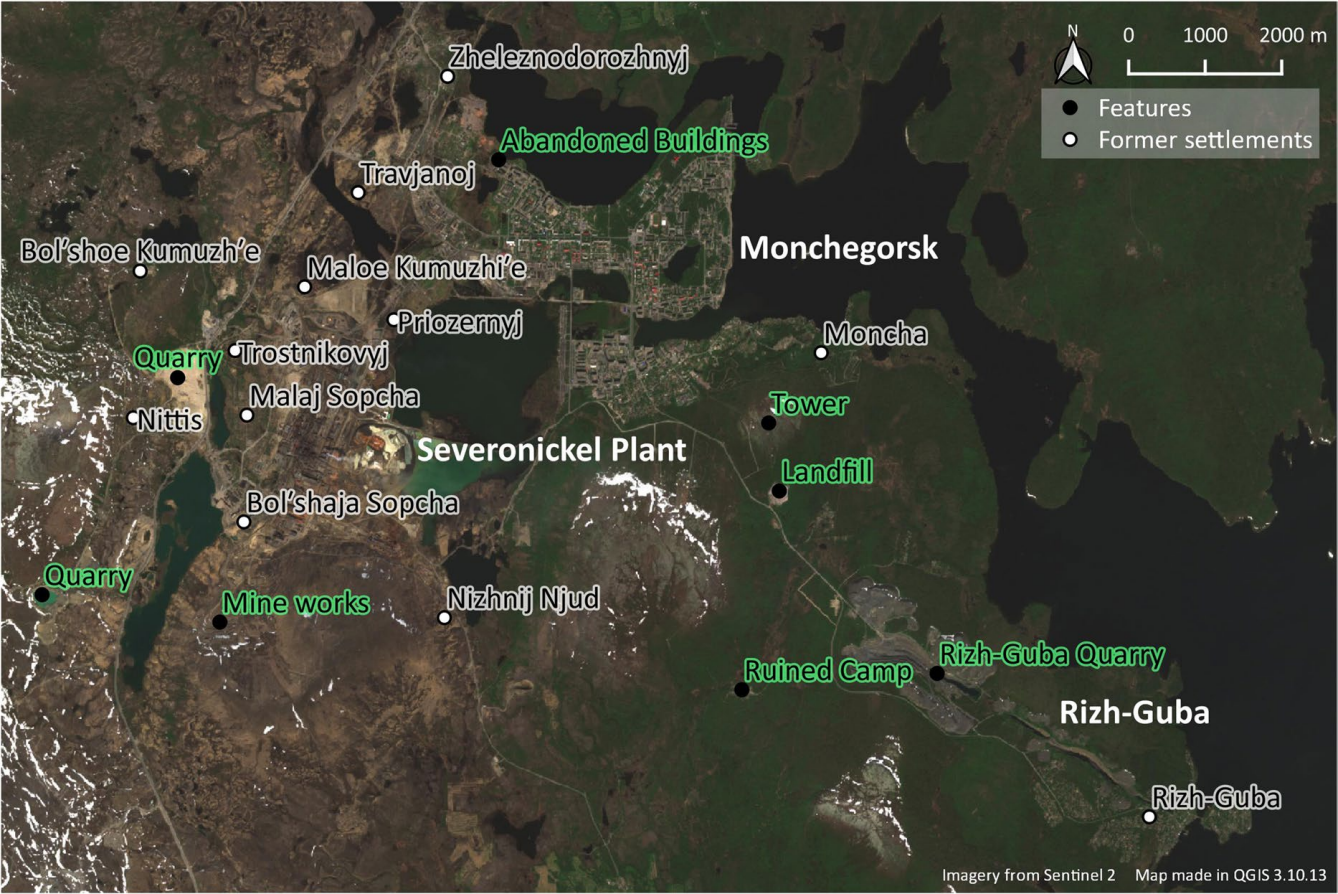
Monchegorsk and its surrounding ancillary impacts and former settlements. Rizh-Guba settlement and quarry are in the *southeast corner*. Note that some former satellite villages lie farther from Monchegorsk and are not shown on this map. (Map by author, 2020.)

The construction of Monchegorsk and its associated processing plant of Severonickel began in 1935 to extract and process the local deposits of nickel, copper, and cobalt (Bruno 2016:179,185–191). The Soviet planners designed the town as the local civic center (*Соцгород* or “Socialist City”) (Poznjakov 1999:23), while around Monchegorsk 16 separate satellite villages sprouted up (Dezhkina 2015) (Fig. 2). While the “Socialist City” provided civic and administrative services as well as housing for the local elites, the villages provided the center with goods and services in the form of workers’ housing close to the primary industrial operations, quarrying, forestry, railroad connection, and collective farming (Dezhkina 2015).

One such satellite village was Rizh-Guba (Риж-Губа). Located 4 km from the “Socialist City,” it provided housing for workers and their families who extracted quartzite from a quarry (also called Rizh-Guba) that was used for the copper smelting process at Severonickel. Operations began in 1938, with 215 people and 13 houses documented in the village that same year (Beljunas 1938; Poznjakov 1999:223). The village expanded significantly after the war. A June 1954 article in the local newspaper *Monchegorskij Rabotnik* (*Мончегорский Работник*, or “Monchegorsk Worker”) celebrated the modern village for having several houses, clinic, school, kindergarten, shop, cafeteria, bathhouse, vegetable storehouse, and all other essential facilities (*Monchegorskij Rabotnik* 1954).

The workers performed early quarrying by hand, focusing on the richest deposits in the southeast section of the formation. Before a road was built to the Severonickel processing plant for mineral transport in 1955, the only access to and from the village and quarry was by boat over Lake Imandra (Poznjakov 1999:224).

By 1975 the quarrying depleted good quality quartzite deposits and extraction moved to the northwestern region of the deposit. Here, the quarry employed more mechanized methods for extraction to keep pace with the expanding production demands at Severonickel throughout the 1970s and 1980s (Poznjakov 1999:224; Bruno 2016:204). These methods employed the techniques developed in mass-destruction mining, including drilling, blasting, and moving massive amounts of rock and reprocessing it to extract the materials considered valuable (LeCain 2009). By 1983 the local authorities resettled the entire village of Rizh-Guba and turned it into a recreational resort (*база отдыха*, literally “base of rest”). At the same time, the area around the village was zoned for dachas (Russian recreational cabins) through the travel facilitated by the 1955 resource road (Kraevedcheskij Portal Monchegorska 2017). Work at the quarry continued until 1993 when production stopped; the quarry was never remediated (Poznjakov 1999:225). Recent discussions surrounding Rizh-Guba, including local news outlets and the municipal plan for Monchegorsk that only marks the site as an abandoned quarry (Institut “Giprogor” [2010]), ignore the area’s industrial past and focus mostly on Rizh-Guba’s importance and status as a recreational and gardening area in the Monchegorsk region.

Most of the other settlements around Monchegorsk also disappeared in the 1970s and 1980s. Soviet authorities relocated and absorbed some of the villages into the larger town while a few others were abandoned after the collapse of the Soviet Union (Dezhkina 2015). Their remains are in various states of preservation—from still-standing husks of buildings to systematic erasure through demolition and subsequent redevelopment. As for Monchegorsk itself, it was particularly vulnerable to the shifts in the political and economic conditions that came with the Soviet Union’s collapse. With the breakdown of the Soviet system and the privatization of businesses, industries that did not shut down required downsizing of the workforce, downscaling of industrial operations, and reduction of company investment in their communities. While smelting operations continue in Monchegorsk to this day, the population has fallen from a peak of 72,500 in 1993 to 42,000 in 2018 (Lukichev 1993:105; ROSSTAT, Federal′naja Sluzhba Gosudarstvennoj Statistiki 2018).

## Western Labrador and Wabush Sand Pits

Russia is not the only place where recent economic and political shifts led to growth and subsequent contraction in northern industrial regions. While the Soviet Union was developing its resource frontier, Canada was engaging in its own northern colonization by offering lucrative opportunities for companies to develop industrial communities in the Canadian Arctic and sub-Arctic hinterlands. While development was slow at first, the postwar economic boom in the United States spurred resource demand, creating dozens of new extraction-based communities in northern Canada (Zaslow 1988; Piper 2009).

**Fig. 3:**
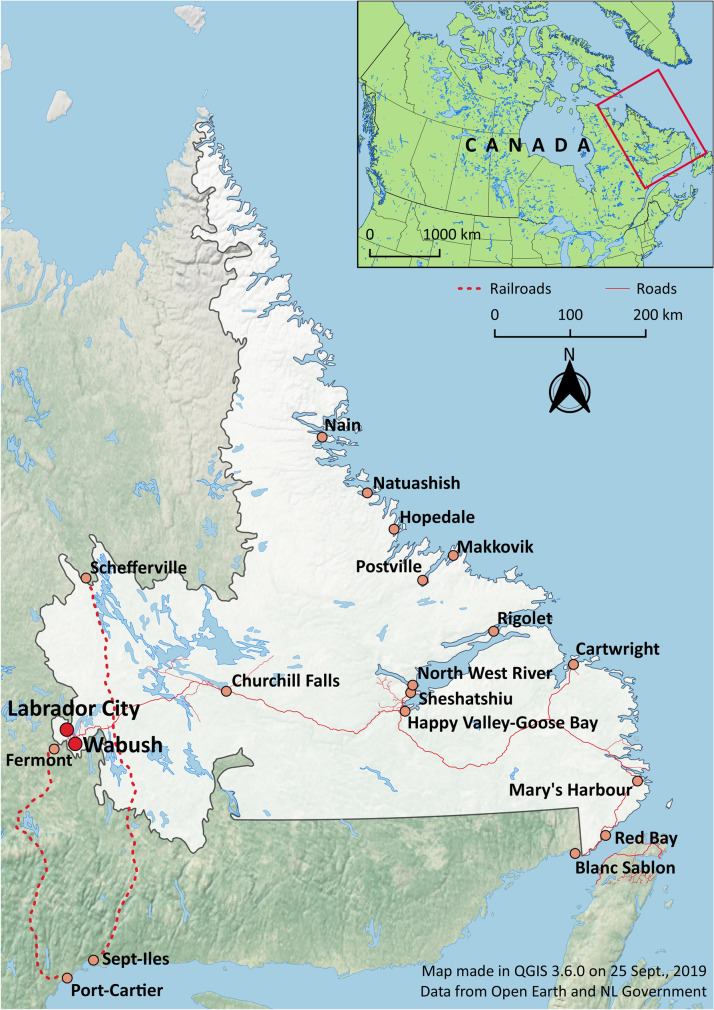
Labrador City and Wabush in Labrador, Canada. (Map by author, 2020.)

One place to experience this rapid industrialization was the interior of the Labrador Peninsula located in northeast Canada (Fig. 3). Much as with the Sámi on the Kola Peninsula, the Innu occupied the interior of the Labrador Peninsula for thousands of years with limited interactions with the Europeans. However, in 1954, the completion of a 580 km long railway into the interior of the peninsula opened the region for iron mining and hydroelectric development. The provincial governments of Newfoundland and Québec, which share the peninsula, encouraged these constructions to make a supposedly empty barren land^1^ profitable for American and Canadian market investment and create a new settled northern frontier (Schoenauer 1976; Ponte and Kowal 2008; Thistle and Langston 2016:274–275).

This inflicted rapid changes upon the Innu who experienced marginalization, resettlement, and the destruction of their hunting and camping grounds brought about by sudden industrialization. While some Innu groups were able to successfully navigate the difficult new realities of an industrial economy (Boutet 2012, 2013), others were forcibly resettled without compensation and their lands flooded by hydroelectric reservoirs built in part for mining development (Loring et al. 2003; Neilsen 2016; Venovcevs 2020). Much like in Monchegorsk, whose name “Monche” derives from the Sámi word for “beautiful tundra” (Bogomolov 1957:76), traces of Indigenous presence are visible in western Labrador on a little peninsula called “Indian Point.” There are scattered written and oral accounts of several Innu families living there in the early 1960s, which included interactions between the first settlers and the Innu along with problematic company practices of supplying them with unlimited alcohol (Maher 1992:5–6; Marcil and Greene 1992:9–11). The mining companies forced the Innu to move by the late 1960s, although the local settlers report observing traces of their cabins for a couple of decades afterward (McLean 1995:56). Recent surface surveys that I conducted in the area were not able to relocate the traces of these cabins.

**Fig. 4:**
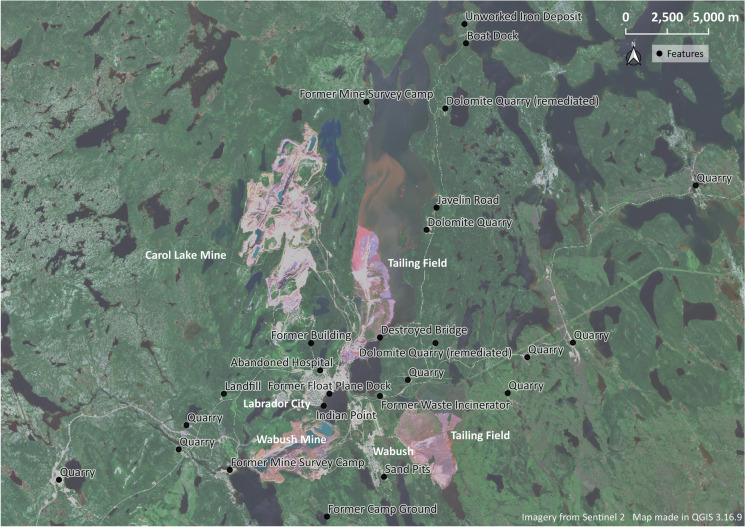
Western Labrador mining towns and their surrounding ancillary impacts. Wabush sand pits are in *center bottom*. (Map by author, 2021.)

In this context, the twin iron-mining single-industry towns of Labrador City and Wabush were founded in 1961 and 1965, respectively (Hilton 1968:53,147; Rompkey 2003:120,124). While the mining companies designed the towns with relatively dense urban cores adjacent to their iron-mining and processing sites, the towns’ development required the construction of a dispersed network of associated industries that included railroad sidings, airstrips, gravel pits, access roads, survey camps, cut lines, quarries, and a dedicated hydroelectric power station (Fig. 4) (Geren and McCullough 1990; Arsenault 1997; M. Harrison 2003; Rompkey 2003:125; Venovcevs 2020).

The mining towns grew rapidly in the 1960s and 1970s, but the optimistic promise of an urbanized northern industrial frontier ended abruptly in the early 1980s with a collapse in the iron ore prices. This led to the closure of two iron-mining towns on the Labrador Peninsula and the reduction of other mining operations including those in Labrador City and Wabush (Bradbury 1983, 1984, 1985). Mining companies that had already started divesting themselves of their nonindustrial holdings since the late 1960s, further cut back on their expenditures and focused attention on restructuring their industrial operations. From their peak in the mid-1970s, the population of Labrador City and Wabush fell from 15,000 and 5,000, respectively (Geren and McCullough 1990:278), to just 9,000 people total in both communities in 2016.^2^ The golden age of Canadian northern industrial city building was over. Much in the way that the collapse of the Soviet Union’s planned economy made Soviet single-industry towns vestiges of a bygone regime, so too single-industry communities in Canada are vestiges of a different political and economic system that no longer seeks to build single-industry communities as a way of northern colonization. All current mining operations favor a flexible labor force through the construction of work camps with a rotating fly-in-fly-out schedule for workers.

Much like in Russia, the area around Labrador City and Wabush is surrounded by active and abandoned ancillary industrial operations (Fig. 4). For the purposes of this article, I want to highlight an area southeast of the town of Wabush colloquially referred to as “the sand pits.” Prior to the town’s construction, this area was marked by a series of cut lines created as part of the extensive drill survey campaigns that took place from 1955 to 1959 to map the iron ore deposits in the area (D. Hynes 1990:7–8). The area was briefly abandoned from 1960 to 1962 as mine construction started, with workers and their families living in a tent camp near the mine (D. Hynes 1990:12–18). That changed in 1962 when the townsite for Wabush started to be constructed.

The townsite lies on the steep rocky western slope of a hill that occupies an approximately 6 km^2^ area between Jean Lake to the west, Wahnahnish Lake to the south and southeast, and the Flora Lake (now Wabush mine’s tailing field) to the east. Given the area’s steep slope, significant amount of fill was required to make the land suitable for building the single, double, and row housing that characterizes Wabush’s “Garden City” design. This design included the need for rich topsoil to create grass-covered front lawns—something that an early souvenir booklet from western Labrador advertised as a good use for the Labrador muskeg (Young [1964]). The extraction for the necessary sand, gravel, and topsoil took place on the eastern and southeastern edges of the townsite, behind the expanding town. Large-scale aggregate extraction continued into the early 1970s when much of the town’s development ceased, leaving behind a series of pits.

Interestingly, no archival references or pictures exist of this activity, and much of the information about them comes from talking to local residents (Jordan Brown 2019, pers. comm.; Gary O’Brien 2021, pers. comm.). As such, the sand pits are essentially “pre-historic”—written out of history. The extraction of aggregate on which the town sits, it would seem, did not warrant a mention in the town’s contemporary advertisements or the subsequent histories, despite attentive focus on the issues surrounding housing and town design (Young [1964]; D. Hynes 1990; Riggs 2019). Even the 1985 Town of Wabush plan, while clearly depicting the former cut lines surrounding the sand pits, only represents the pits as a series of treeless depressions (D. W. Knight Associates 1985).

After the main construction phase, the town of Wabush continued to use several of the pits to harvest sand for road maintenance in wintertime, but this too seems to have stopped in recent years (Gary O’Brien 2021, pers. comm.). The last attempt to put this area toward extractive use was in 2010 when the Wabush town council denied an application for further sand quarrying. Overall, the sand pits were left as they are. It could be postulated that if the town of Wabush continued to expand, the sand pits would have disappeared under residential subdivisions. However, stagnation and population decline from the 1980s onward prevented that redevelopment. Recent rezoning has also ensured their survival into the future as the 2019 Town of Wabush zoning regulations subsumed much of the area into an expanded watershed protection zone. The rest of the area is zoned as “open space” (Stantec 2019).

Today, the pits lie as a “a space left over” (Andersson 2014), beyond the concerns of historicity and municipal planning. However, they still exist physically and within the local imagination. In the recent exhibit by the western Labrador artist, Tanea Hynes, images of the sand pits appear twice (T. Hynes 2021:11,18). While they are without caption, one of them—an image of car wrecks discussed later in this article—is described briefly on a separate page under “worth noting” as simply “wabush sand pits” (T. Hynes 2021:84).

## Vestiges

As the examples above and Figures 2 and 4 demonstrate, industrial development in these mining towns was not limited to the urban boundaries of the towns themselves. Rather, they are surrounded by a landscape filled with ancillary operations. Many of these are the result of the economic growth and construction that took place during the second and third quarters of the 20th century. Subsequent economic decline and contraction led to the abandonment of many of these operations, leaving behind an assemblage of features that litter the peripheries of these industrial towns. To grasp and constructively talk about these landscapes, I propose to approach these features as vestiges of ancillary impacts of resource development.

The word “vestige” has enjoyed increasing popularity in archaeological literature in recent years. Scholars have used the term in a wide variety of contexts such as to describe atomized traces of human figures imprinted as shadows on the ground from an atomic bomb explosion (Domanska 2017), or objects that have been detached from their intended purpose and function such as hyperart (Farstadvoll 2019c), nonabsent material presences of the past (Shanks 2012:133–136), untimely anachronisms (Lucas 2015:7), and “pluritemporal” objects of material memory (Olivier 2011:4–8).^3^

The problem with these uses is that the concept of vestige has remained largely undefined, and most archaeological applications take slightly different meanings. For Michael Shanks (2012:134), for example, the key point of vestiges is their ability to interrupt the current moment through a haunting presence of that which is no more. In this sense, vestiges are active future-facing elements of a nonabsent past that reemerge as uncanny things: that which layers of history should have concealed reemerges and intercepts the perceived linearity of time. Whereas for Ewa Domanska, vestiges are passive objects from the past—a chemical fusion of atomized organic remains with nonorganic stone as a result of an atomic bomb, which highlights the more-than-human realities of the Anthropocene epoch (Domanska 2017). Meanwhile, Stein Farstadvoll (2019c) uses the term vestige to celebrate decontextualized objects for their meaninglessness, dislocation, weirdness, and defiance of human expectations pointing toward the fragmentary composition of landscapes.

The only scholar to explicitly define the word “vestige” is Laurent Olivier. For Olivier, vestiges are relics or prized possessions that witnessed a vanished history but remain in association to memories of those events and people who have passed. The association of dynamic meanings with objects is what turns objects into vestiges. As such, vestiges are the fluid material manifestations of the past in the present. However, rather than simply serving as carriers of material memories—indexes to be unlocked through habitual reengagement with objects and landscapes (Jones 2007) or reminders of disjointed nonabsent pasts that pull us toward habitual mnemonic refamiliarization upon encounter (Olsen 2013)—the vestige is free to evolve and becomes something else outside of and separate from human engagement with it. Much like the plants in Farstadvoll’s (2019a) garden, vestiges grow on their own accord with meanings reinvented rather than recalled. Thus, as carriers of material memories, vestiges give little guarantee that the meanings are the same in the present as they were in the past or that they will be same from person to person, let alone to nonhuman persons. The stripping of meanings, creation of new ones, and the entwining and blending of meanings and associations is what gives vestiges pluritemporality that undoes sequential and linear chronological continuity. Objects only stop being vestiges and become artifacts when memory has been lost and they cease receiving new meanings (Olivier 2011:6–8). For Olivier, remains and artifacts are retrospective and stuck in the past while vestiges are evolving and multitemporal.

The growing ubiquity of the word vestige may have something to do with it signifying a disarticulated, enduring fragment from the past that has lost its broader context and yet is persistent and remains tied within a meshwork of shifting memories and meanings. Implicit in many of these discussions is the fact that the vestige is still recognizable*—*a shape of a human figure, a lacquered wooden box, or a plastic road stake—and it is that recognizability that carries the haunting and uncanny sensations and the abilities to evoke involuntary memories. An object would not have the same power if it was too small or too disarticulated from what it once was or could have been—a piece of lacquered wood or a disintegrated plastic fragment. However, recognizability in the context of a vestige almost guarantees that that recognition will not be the same as when it was in its original context. The objects in Olivier’s family reliquary are different to Olivier than they were to the family members they belonged to. Still, they are recognizable to Olivier because of the mnemonic associations he has to the objects.

Recognizability of a vestige poses a few questions—recognizable by whom, in what ways, and when does a vestige become unrecognizable? These questions are compounded by the fact that most scholars cited have backgrounds in archaeology, a discipline that excels at recognizing faint traces of objects and landscapes. While this expert-dependent definition of vestige could be of value to some people, especially heritage planners, I want to advance a posthuman alternative in this article—especially in the current case studies that approach vestiges on a landscape scale. Taking inspiration from Dag Andersson’s (2014) discussion on the ontology of leftover spaces, I posit that an object remains a vestige as long as it is recognizable to itself. Meaning that, as long as an object is self-contained and self-complete, as long as there is a certain degree of internal cohesion within the object, it will maintain its vestigial essence to act as a conduit for involuntary associations and meaning making among the human and nonhuman others who encounter it.

As such, it would be useful to return to the etymology of the word “vestige,” which originates from the Latin *vestïgium* meaning “footstep, footprint, trace, mark” (*Oxford English Dictionary* 2020). Central to this definition is the unseen, former physical act whose consequences cut across time and are encountered in the past, present, and future. The focus on the self-contained material consequence has great utility for studying the remains of the resource extraction industry that left a broad assemblage of material consequences within the landscape where resource extraction has existed. Furthermore, and in line with the work of Olivier, Shanks, and others, the material consequences are not just passive elements of former human action but rather remobilizing active agents that shape and reshape the futures to come. It is through this lens that I return to the sites of Rizh-Guba and the Wabush sand pits to see just how these vestiges of ancillary impacts of resource extraction play out in the present.

## Surveying Vestiges of Ancillary Industrial Impacts

Much of my fieldwork in western Labrador and the interior of the Kola Peninsula has followed an archaeology-of-surface-assemblages approach, similar to other recent contemporary archaeology research (R. Harrison 2011; Pétursdóttir 2013; Farstadvoll 2019b; Olsen and Vinogradova 2019). The work consists of site visitation, pedestrian survey, photo documentation, GPS recording, and notetaking to see how the single-industry mining towns and their peripheries articulate themselves in the present.

While I explore many of these places on foot, there are drawbacks to solely relying on this approach given that contemporary industrial operations create massive features that limit the embodied understandings that characterize most archaeological survey techniques. As supplement, I have been using satellite imagery available from Google Earth Pro and ESRI to understand these large objects in a manageable format. While there are drawbacks in relying on commercial, proprietary software systems (for discussion, see McQuire [2019]), they do provide accessible high-resolution imagery to ascertain the nature and scope of the material remains of ancillary impacts. In addition to that, I employ declassified American spy satellite imagery for the Kola Peninsula and historical orthoimagery for Labrador to examine how the regions in question appeared in the late 1960s/early 1970s.

Preliminary ground-based fieldwork took place at both sites during the spring and summer of 2019, focusing specifically on documenting sites of ancillary operations. While the global COVID-19 pandemic prevented revisitation and further field research in Russia, an extensive round of fieldwork took place in Canada during the summer of 2021. The time slots were sufficient for site visits of many ancillary industries highlighted in Figures 2 and 4, allowing me to record their present postindustrial conditions. Additional research was carried out in both the local and regional archives at both locations through COVID-19; pandemic guidelines limited the number of interviews that could be conducted. The subsequent discussion draws specifically on Rizh-Guba and the Wabush sand pits since they represent the most complicated examples of ancillary impacts of resource extraction in my study areas.

### Rizh-Guba

**Fig. 5:**
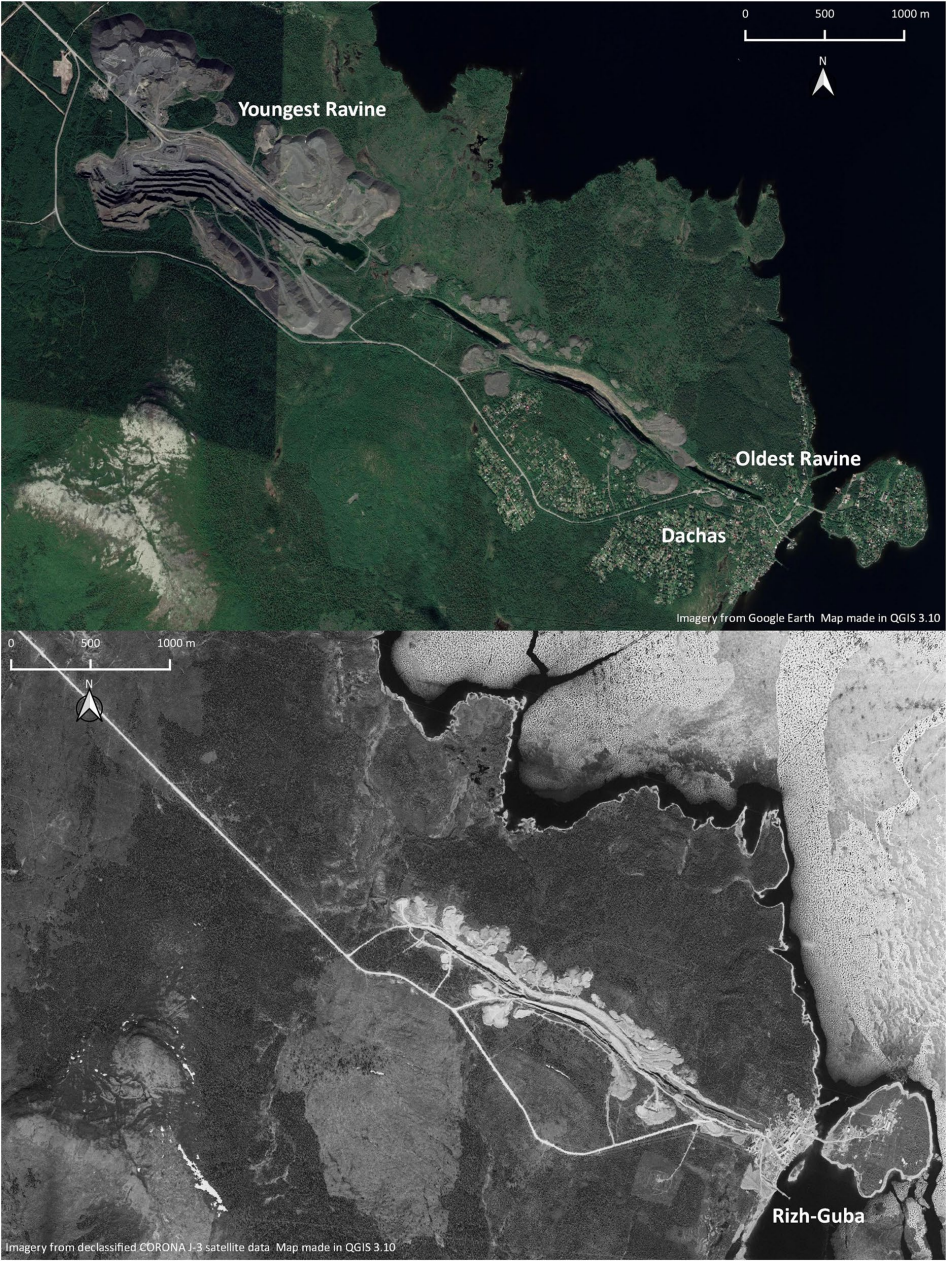
Rizh-Guba quarry on the shores of Lake Imanda in 2019 (*above*) and same area in 1971 (*below*). (Map by author, 2021.)

Seen from satellite imagery, Rizh-Guba appears as a linear feature consisting of four deep, narrow, and long ravines that stretch southeast to northwest from the shore of Lake Imandra (Fig. 5). Taken together, the ravines are almost 5 km long. Abutting these channels are numerous piles of waste rock that can reach over 20 m high and stretch up to 500 m across. The feature has a footprint of approximately 2.8 × 2.8 km.

Approaching this feature from the ground in July 2019, I observed significant horizontal variation in the feature relating to its abandonment and subsequent reuse. The first area encountered as one travels southeast from Monchegorsk is the newest section that operated from 1975 to 1993. Despite operating for only a third of the quarry’s 60-year history, this section represents two-thirds of the quarry’s total size (Fig. 5). This imbalance is explained by the mechanized method of extraction employed at this part of the site and the overall acceleration of production at the Severonickel plant in the 1970s and 1980s leading to massive environmental degradation in the surrounding area (Bruno 2016:208)

**Fig. 6:**
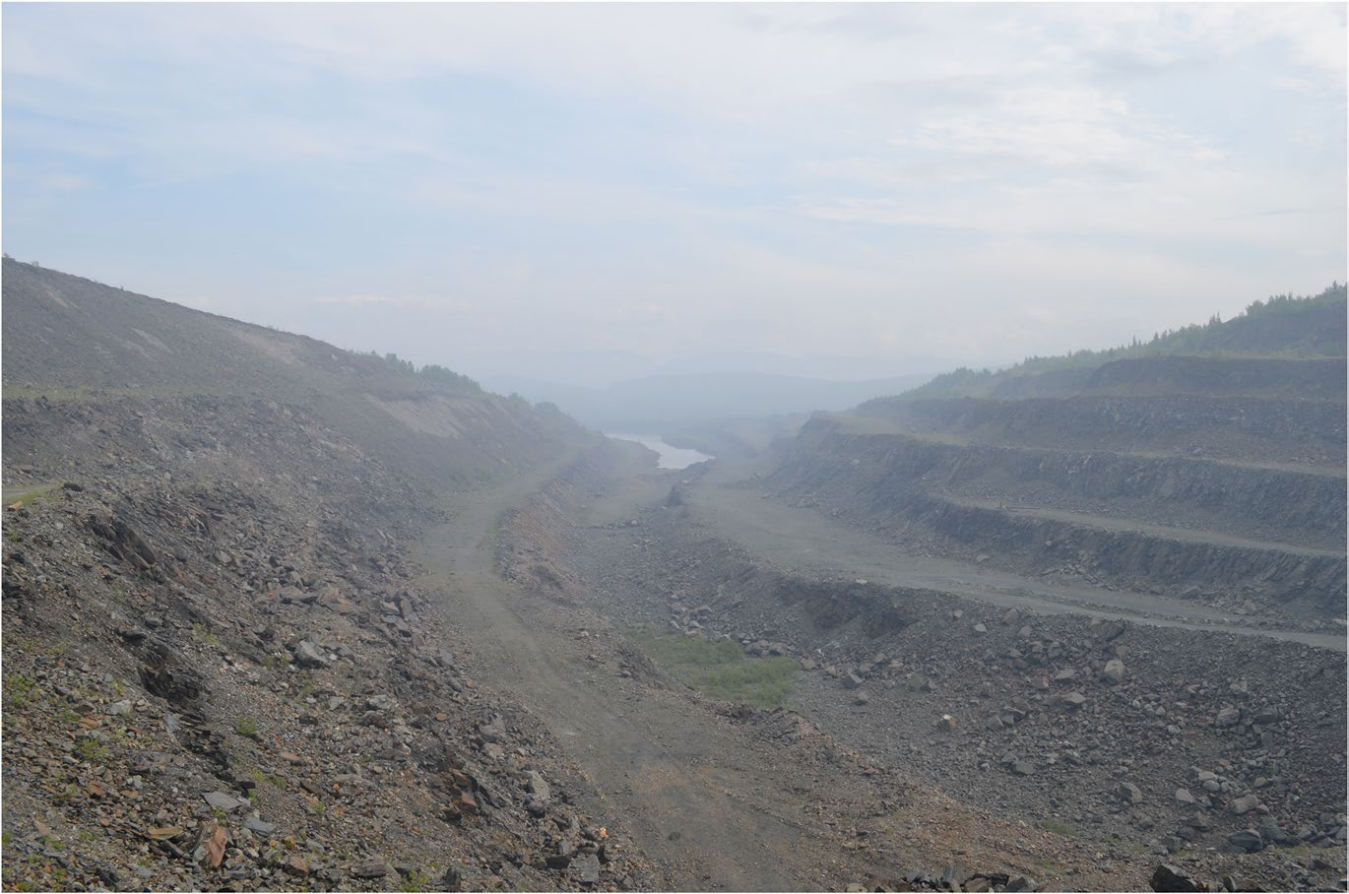
Northwest corner of the Rizh-Guba quarry. (Photo by author, 2019.)

Little material culture remains from the previous quarrying operations. It is mostly limited to a crumbling wooden staircase and the foundations of a concrete ramp used for loading quartzite. All the usable machinery and infrastructure was salvaged or removed, with most of the buildings demolished with the exception of a small, one-story building that contained discarded packing from explosives. The most obvious evidence of industrial activity are the mountains of waste rock and a large open pit mine that remains as a spectral expression of the extractive operations that took place at the site (Fig. 6). While tiny by the standards of open-pit mining, I could not help but feel dwarfed by the barren landscape of mining terraces and waste pits. Even the evidence of post-abandonment human activity was scanty, consisting of a short earthen embankment at the entrance of the quarry to prevent vehicular access, scattered heaps of illegally dumped garbage, a couple of stray tires, and a pile of rusting paint cans and other hazardous household waste.

The feature changes in the southeasterly direction, becoming older and more overgrown. In the center section that operated approximately in the 1950s through 1970s, thin trees have begun the gradual revegetation process of the ravine slopes and waste rock piles. The area is also mostly devoid of human artifacts, though at the time of my visit I observed a makeshift bench that someone built by putting a couple of planks over two worn-out armchairs. It overlooked one of the more overgrown ravines. Next to the bench was a fire pit and a collection of plastic plates, pop bottles, and beer cans in the bushes nearby. This location highlights the informal domesticity that takes place in locations scarred by industrial activity. The act of making a formally blasted area homely reoccurs throughout my study in couches, makeshift fire pits, and other furnishings documented throughout the fieldwork. The pull of turning former industrial landscapes into hangout spots can be explained in two ways: the aesthetical draw of large open ravines, regardless of their anthropogenic origins, and the fact that unregulated brown fields create open spaces for human creativity (Edensor 2005a, 2005b), at least in places that feel relatively safe (González-Ruibal 2017:143).

The engagement with the boundaries of this quarry stands in contrast to the engagement of the footprint of the quarry itself. Surveying one of the more revegetating piles of waste rock, the view presented a beautiful overlook at Lake Imandra but there was little sign of former human presence on top of the waste rock plateaus—only one cigarette butt and two pieces of bottle glass served as evidence for recent human visitation. However, my vantage point revealed that some dachas pressed right up against the walls of the waste rock piles, showcasing continued engagement with this hard and durable industrial past, one that pushed at the boundaries but did not transgress upon the feature itself.

More dachas dominated the area around the southeast corner of the quarry and the former workers’ village. As typical, the dachas are highly personalized, built in vernacular styles, often by the occupants themselves and often representing the occupants’ own professional or individual interests. Only 8 buildings, including the local store, have survived into the present compared to almost 60 structures depicted in the 1971 satellite photo; the rest have disappeared and been replaced by dachas. Some of the worker-village garden plots have also survived and are now maintained by dacha residents.

**Fig. 7:**
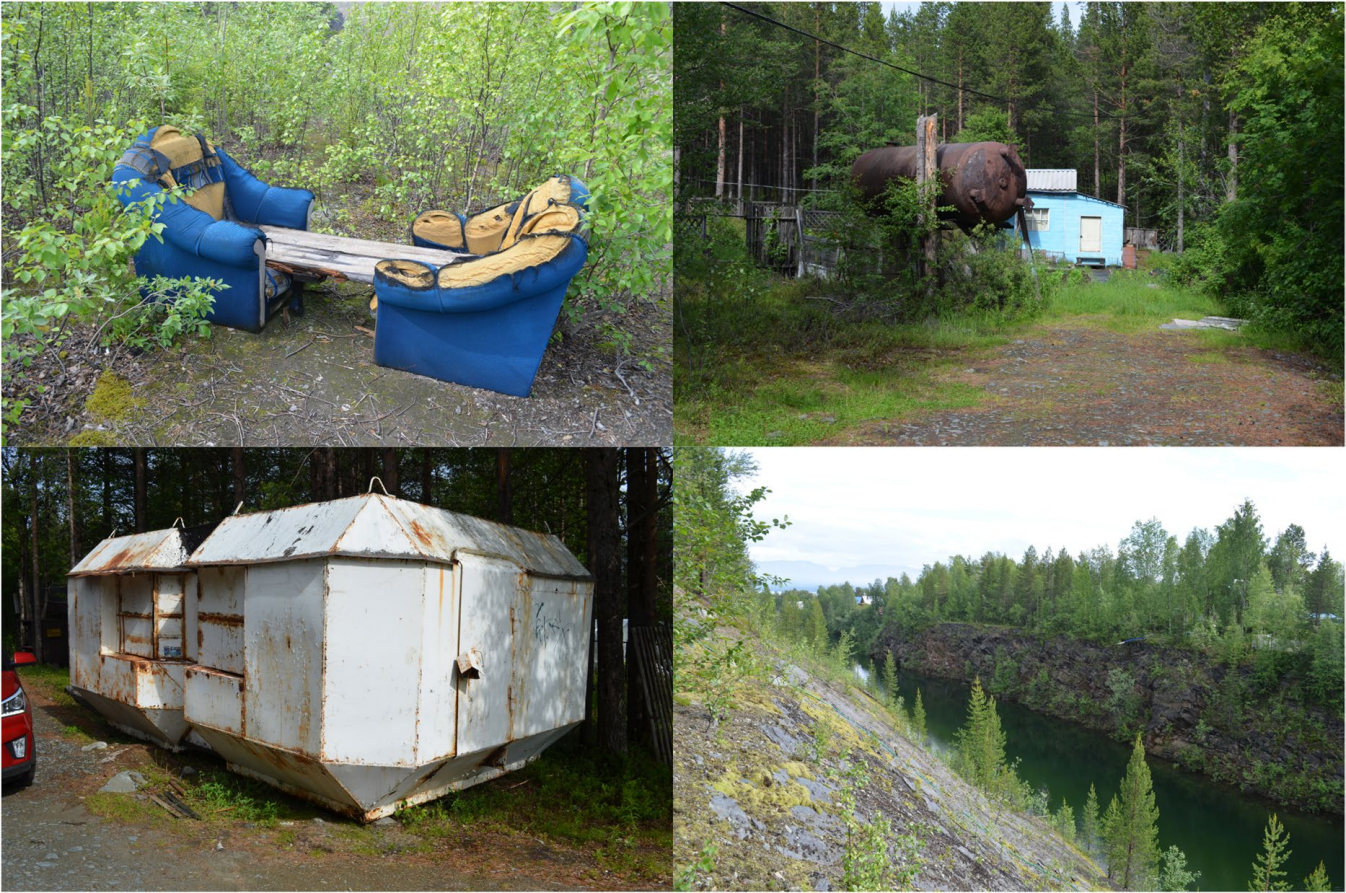
Southeast corner of Rizh-Guba: bench made from two arm chairs (*top left*); reused water tank at a dacha (*top right*); reused rock bin (*bottom left*); and the oldest part of the quarry, now being used as a water source (*bottom right*). (Photos by author, 2019.)

In total, the dachas cover a 2.4 km^2^ area and often abut former quarry workings such as the ravines and the piles of waste rock. Residents commonly reuse old industrial equipment like old fuel tanks, rock bins, and construction trailers for personal use while also drawing water from the flooded quarry ravine (Fig. 7). Much like with their relationship to waste rock piles, the residents of the dacha village do not explicitly occupy the footprints of the industrial vestige around which they dwell. Rather, their interaction takes place at the boundary through utilization of the feature’s affordances.

Dachas present an interesting place of tension in this space. As previous research has demonstrated, residents of the Russian north often use the word “dacha” (*дача*) interchangeably with the word “nature” (*природа*), implying that they consider them to be one and the same (Bolotova 2012:663–665, 2014). They are vital parts of recreation, domesticity, and—especially during times of difficult economic conditions—survival. Thus, one can argue that in the afterlife of Rizh-Guba part of the industrial area has become something akin to “nature.” This is further emphasized by the quarry’s significance as a water source; instead of carrying material memories of extraction via heavy metal toxins (see LeCain [2014]), the materiality of the crater surpasses its industrial preconditions to afford life-giving properties.

The fieldwork and history at Rizh-Guba reveal the fact that despite discontinuation and resettlement of the workers’ village and the shift and decline in extractive operations, human engagement in the area expanded instead of shrank. As the trace left by the quarrying has not disappeared, the footprint of the dachas expanded far outside the confines of the workers’ village.

### Wabush Sand Pits

**Fig. 8:**
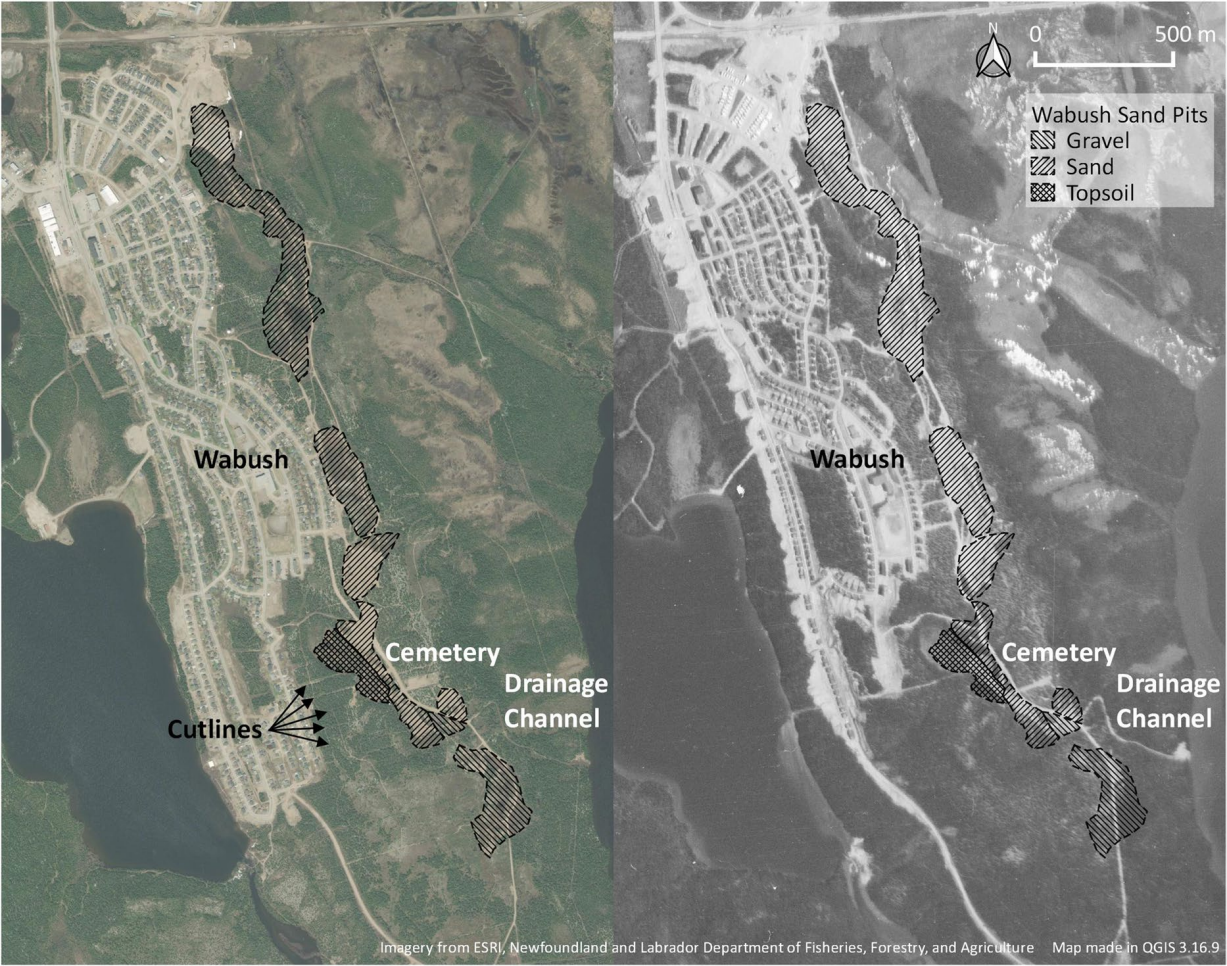
Wabush sand pits in detail as seen in 2019 (*left*) and same area in 1969. (Map by author, 2021.)

Similar to Rizh-Guba quarry, the fieldwork at the Wabush sand pits revealed a multifaceted, evolving landscape that was formed by industrial operations but that has not stopped evolving since then. A 1969 aerial image of the region depicts the sand pits in the middle of their extractive life with four active areas directly east of the then-growing mining town (Fig. 8). The aerial image also reveals a small wetland south of the pits being drained into Wahnahnish Lake and another, smaller clearing further to the southeast. According to a local informant, the wetland was drained for topsoil harvesting for the town’s landscaping while the clearing contained a rock crusher for road gravel (Gary O’Brien 2021, pers. comm.). The former cut lines recorded in the 1985 town plan are also partially visible on this imagery as straight lines going through the sand pits.

Since 1969 sand harvesting shifted southward to new areas, while gravel and topsoil harvesting expanded. Seen today, many of the northern sand pits that were utilized in 1969 have become overgrown while more recent pits remain clear of vegetation. Former cut lines have generally disappeared, although the durability of some has been enhanced by their continued use as trails. Fieldwork in the area confirmed these observations by documenting the reuse and widening of some former cut lines for recreational use and the disappearance of most due to revegetation. In total, the sand pits are approximately 2,800 m long by 125 m wide at their greatest extent (Fig. 8).

**Fig. 9:**
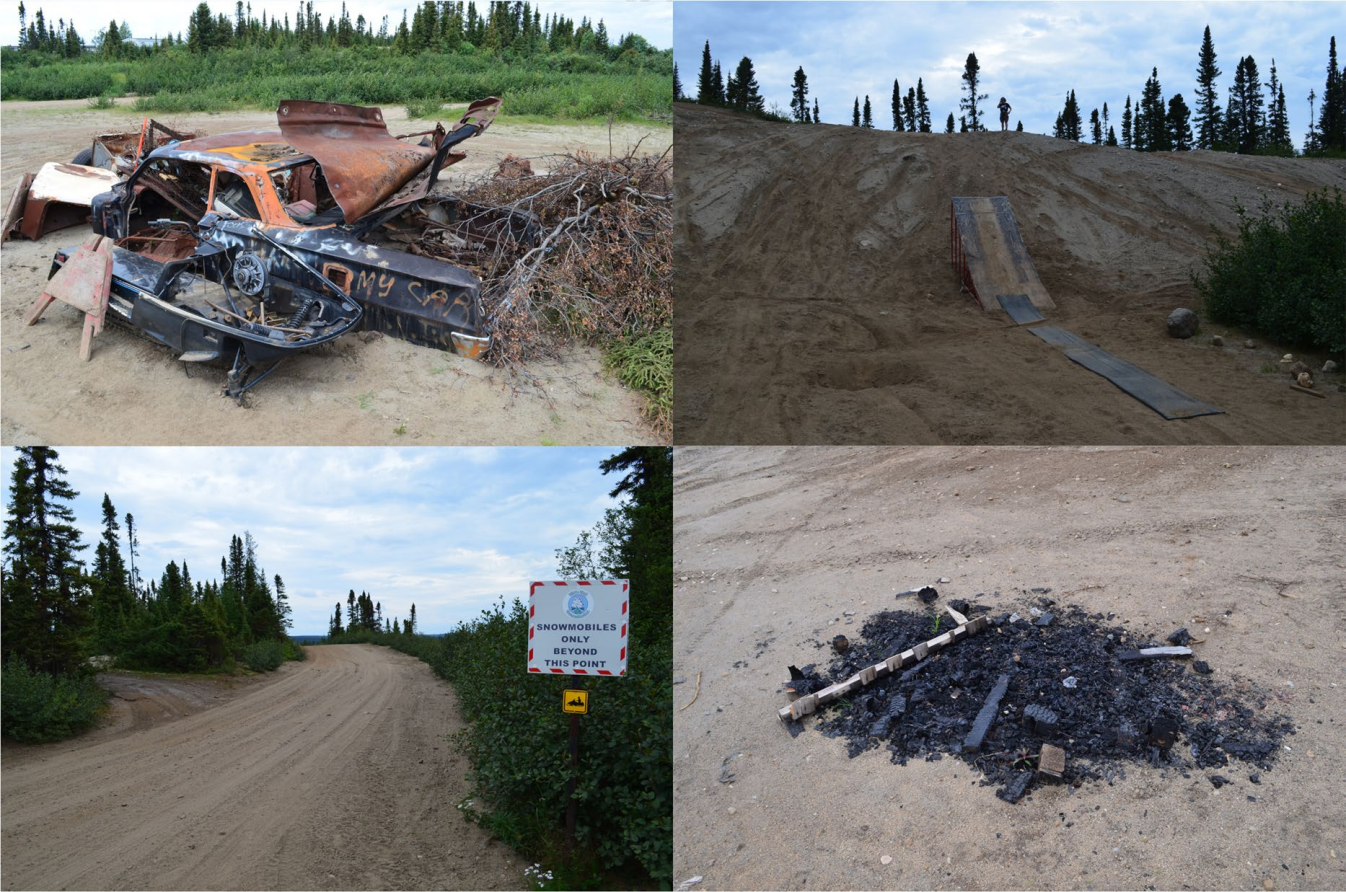
Human use of the Wabush sand pits: vehicle wreck that disappeared after 2019 (*top left*); multicomponent jumping ramp (*top right*); snowmobiles-only road sign (*bottom left*); and an example of a relatively recent fire pit (*bottom right*). (Photos by author, 2019 and 2021.)

Over the years the area also witnessed Wabush’s ski hill, which briefly operated in the 1970s; a ball field, established in the 1970s on the north side of the sand pits; and cemetery, dating back to the founding of the townsite. Until the 1990s, access to the cemetery was through the sand pits themselves. While a new road was constructed to provide access, the old road remains and is colloquially referred to as “sand pit road.” Much of it has shrunk down to only accommodate snowmobiles and all-terrain vehicles (ATVs) and does not receive any maintenance. In the winter it becomes part of the provincial snowmobile system (Fig. 9).

**Fig. 10:**
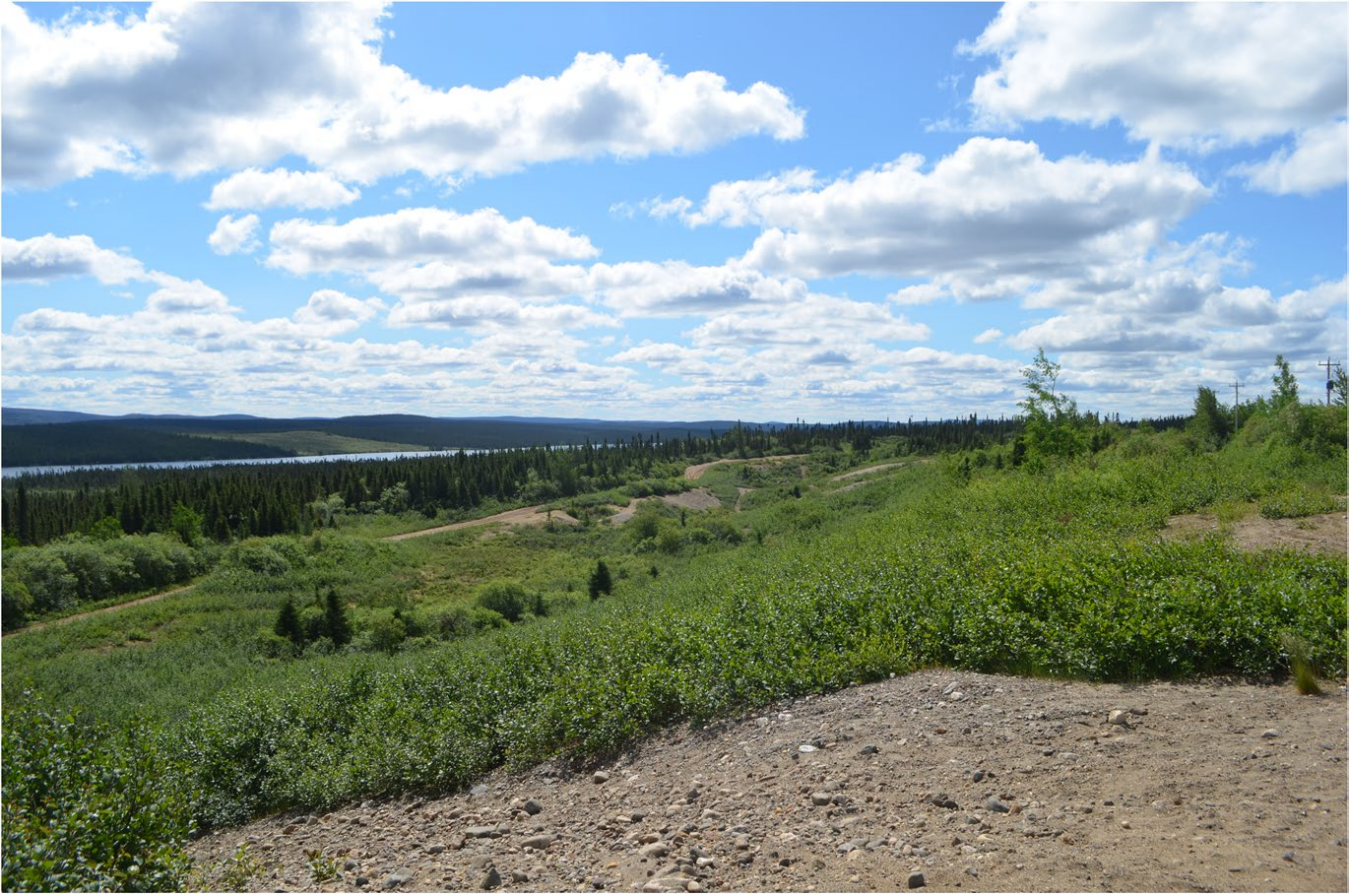
Overgrown sand pit near Wabush. (Photo by author, 2019.)

My surveys of the pits took place in August 2019 and July 2021. During the visits, I observed how much of the original pits have been overgrown by speckled alders (*Alnus incana rugosa*), a virulent Labrador auto-rewilder that quickly takes over road edges, gravel pits, abandoned lots, and other outlying spaces (Fig. 10). Meanwhile, the nonrevegetated areas have become informal places for recreation.

**Fig. 11:**
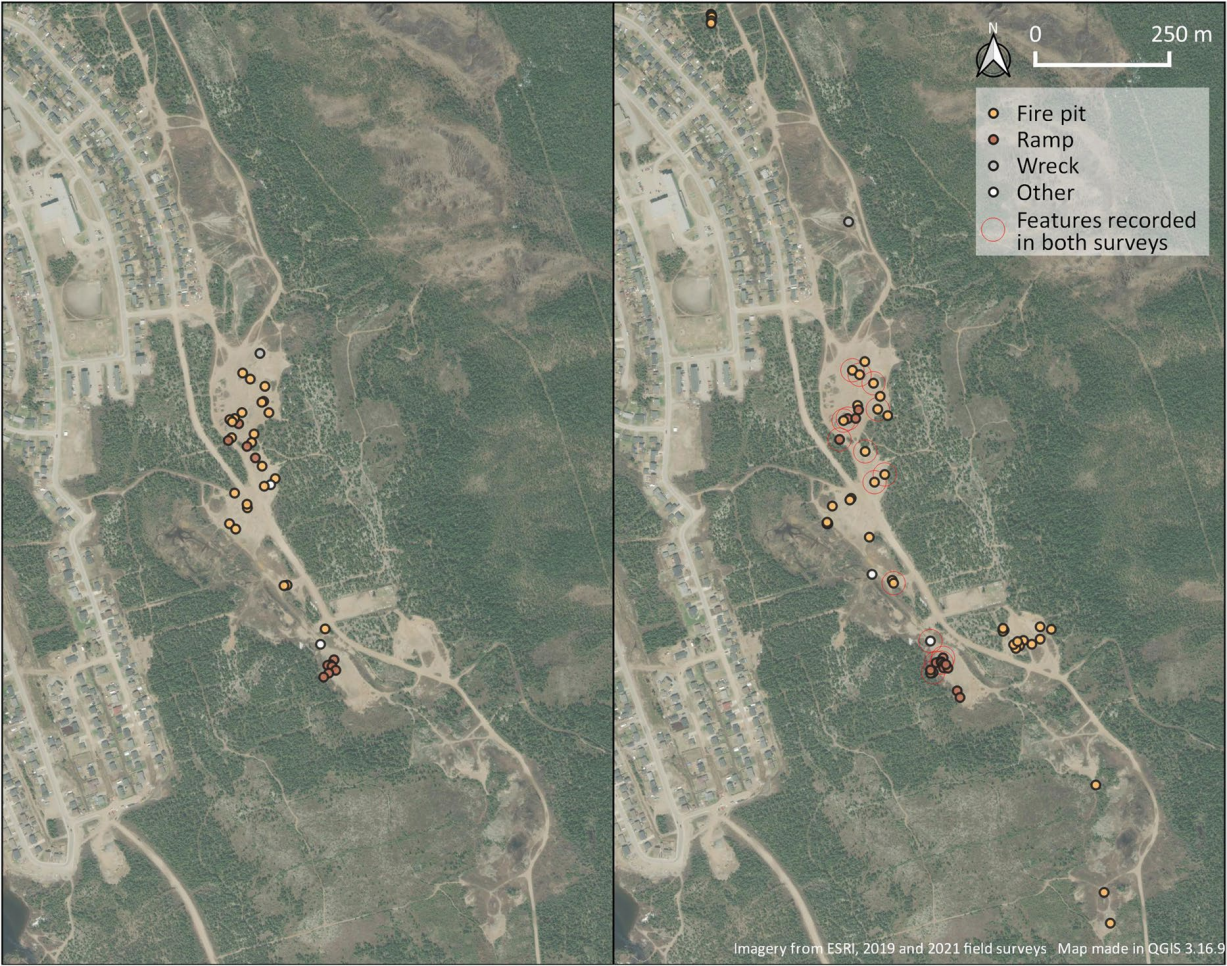
Results of the 2019 (*left*) and 2021 (*right*) surveys. (Map by author, 2021.)

During the 2019 survey, I focused mostly on the nonvegetated areas of the sand pits, recording 24 separate instances of fires (labeled as “fire pits”), 10 jumping ramps for dirt bikes and ATVs, and 3 different vehicle wrecks (Figs. 9, 11). In 2021 this work was expanded to encompass the entire area; all ATV trails (former roads for hauling aggregate) were field walked, and the nonvegetated areas of the sandpits were surveyed at 5 m intervals. The revisitation recorded 42 fire pits, 16 jumping ramps, and one snowmobile wreck.

The work revealed that young people were largely using the area for recreational purposes. This was not a new development as several older informants told me that they used to play around in the area in their youth. While they had memories of this place, however, they noted repeatedly how much the area has overgrown since their youth, as if the plants themselves were signifiers of time in the absence of any visible material culture besides the outlines of the excavated craters themselves. For my informants, the sand pits are a place of memory, though one with fading recognizability due to rewilding. Likewise, these memories were associated with drinking and riding around rather than the industrial activities that took place in these locations.

The second survey of the sand pits also documented fluidities and continuities around the material culture of the area. Some fire pits recorded in 2019 were not present in 2021, having been absorbed by the sandy soil. Ashes of several other fire pits were starting to scatter and dissipate into their surroundings. Meanwhile, jumping ramps presented interesting observations as well. Some of the ramps were made of wood or wood-and-rubber composites, with the rubber recycled from conveyor belts in the iron ore crushing and concentrating plants in the mines. These jumping ramps are more durable with the same ramps being recorded in both surveys, though some were starting to show their age. However, most of the ramps were made of piled sand and some of the largest ramps seen in 2019 were almost completely eroded two years later. The use of sand ramps further indicates the shifting, impermanent nature of the area. New sand ramps could be built as old ones eroded back into the ground as part of a dynamic practice of maintaining informal recreational infrastructure for dirt bike and ATV users.

In 2019 I assumed that the most durable pieces of material culture in this area would be the car and ATV wrecks. Out of the three recorded in the first survey, one consisted of a burned-out ATV that had rolled over on its side while two were a highly disassembled car and snowmobile found together with their metal parts strewn about in a tightly concentrated area. This concentration also contained other waste such as brush cuttings, two modern stoves, and two “Town of Wabush” traffic barriers (Fig. 9). This concentration is featured in the aforementioned art exhibit by Tanea Hynes, highlighting its prominence as part of the Wabush sand pits (T. Hynes 2021:11). Yet, to my surprise, all the vehicle wrecks recorded in 2019 had disappeared by 2021. This indicates that the sand pits undergo episodes of informal cleaning (informal because the Town of Wabush takes no responsibility for the pits today), which means that even small landmarks, such as the wrecks memorialized by the art of Tanea Hynes, disappear as the area undergoes successive changes. In total, only 16 features recorded in 2019 were present in 2021: 9 fire pits, 6 ramps, and 1 ductile iron pipe.

The elements that proved durable in the sand pits were the landscape alterations themselves. In addition to the pits and the afterlives of former cut lines, the field research documented the remains of the bog used for topsoil harvesting. During fieldwork, the bog did not appear so much as a wetland as it did a low-lying depression crisscrossed by ATV and snowmobile tracks. The remains of the ductile iron pipe and ditch that drained the wetland ahead of extraction were also present, having become overgrown, silted-in, and partially buried. The pipe is the only real artifact from the extractive use of the area. Meanwhile, there were no traces of the rock crusher on the southern end of the sand pits, although the area around it contained plenty of glacial till that was extracted for gravel.

Finally, during my surveys, I observed several people utilizing the area. In addition to youth on dirt bikes and ATVs, several older people used the area for walking or riding bicycles, showing the multipurpose recreational use of the sand pits. While Wabush has several recreational trails and a town park, this unstructured area seems preferred. One advantage that the sand pits have over other recreational areas is that they are one of the few places in town where the mine and the mine workings are not visible. It could be postulated that in a community where the town and the mine are literally facing each other, a large area away from industry that allows for informal play and recreation creates a powerful “node” (Gordillo 2014:21) for frequent reuse and revisitation—a place to be in nature but also in a place that affords new meanings, memories, and attachments in an ever-evolving palimpsest.

## Discussion

Even though Rizh-Guba and the Wabush sand pits may seem spatially and chronologically worlds apart, they both supported industrialization of their respective regions and both continue to persist and expand in the present. It would be improper to call these places “artifacts” of mining developments (following Olivier’s conceptualization of the word) since they are actively evolving objects that have retained a part of their original essence even though the broader political and economic shifts in the late 20th century have decontextualized them from their original intended purpose. This survival is because planning and development interests have—at least in part—forgotten these features, allowing them to escape much of the discourse regarding remediation and heritagization that often surrounds former industrial spaces. The lack of active human concern is perhaps not surprising as both the mines in Wabush and the refinery complex at Severonickel have in recent years struggled for economic survival and environmental accountability (for some examples, see Genge [2009], Goodyear [2012, 2015], Moiseenko et al. [2018], Britskaya [2019], and Nilsen [2019]). What planning intervention that did take place has so far promoted human presence and interaction with these landscapes but not in their original ways. At the same time, vernacular reuse illustrates how the material affordances of industrial vestiges can be unequivocally different from those that dominated their active use, highlighting the difficulty in pinning original meanings associated with objects and places.

There is a tension here. On one hand, one can observe a freedom to escape and grow beyond human planning and intentionality. While on the other hand, there is also a dependence and active reuse of the past where elements of former industrial operations sustain themselves through new roles. As I have explored elsewhere (Venovcevs 2021), there is a past dependency and involuntary habit memory that comes across particularly strongly in northern single-industry mining towns where old materialities do not disappear but remobilize to serve new purposes in each successive present. While these new purposes are creative, vibrant, dynamic, and, at times, transgressive toward original human intentions, they are still circumscribed by the things’ own material affordances. This can be seen here with dachas utilizing a quarry that inadvertently filled up with potable water or with motorized recreational vehicles that maintain the footprint of a sand pit through use that discourages revegetation.

This feeds into the pluritemporal aspect of vestiges, meaning that they can simultaneously evoke multiple meanings at multiple times. As observed in the case studies presented here, associations with vestiges differ between industrial and postindustrial use, across succeeding instances of postindustrial use, and even geographically over time as the whole evolves into multiplicities. Those fragments in turn further surpass their previous historical boundaries and shoot off on different trajectories expanding in an ever-deepening mosaic. On a long-enough timeline, it would be impossible to think of a landscape vestige as a single, unseparated whole. This fragmentation speaks to the temporal unevenness previously observed among the afterlives of industrial operations that emerge in situations where the character of former industries is poorly defined, recognized, or agreed upon (Storm 2014).

Fragmentation and fluidity highlight the illusiveness of vestiges to stay pinned to a single identity. Most people I talked to, along with newspaper and documentary accounts, did not readily connect to either Rizh-Guba or the Wabush sand pits as places of extraction. Rather their connections to these places are those of dwelling, rest, and recreation, along with associated activities of fires, riding, drinking, access to nature, and, in Wabush’s case, entrance to the cemetery. While there are still people alive who worked in extraction in both of my case study areas, the current associations are distinctly different from past ones. Their ascription as industrial, albeit involuntary and contemporary, heritage comes from me as an archaeologist and a scholar who has tracked their developments and post-use lives rather than any local recognition to their historicity. My argument here is not that this historicity should be acknowledged or celebrated, but rather merely heeded to understand how the dispersed remains of ancillary operations outlive their uses in the service of sites of industrial production.

This gets us back to the topic of recognizability. The recognizability of vestiges by most human observers becomes fainter and fainter as the industrial vestiges grow vaguer over time, as highlighted by the disconnect between peoples’ association of recreation with these places and my draw to them as industrial heritage. While the identification of paleolithic stone quarries by archaeologists suggests that they would always be recognizable at least to some human agents trained in that task, their internal self-recognition implies that fragments of vestiges will always evoke meanings while they remain self-contained and self-complete. While traces and marks remain in the landscape, human and nonhuman agents will always have to contend with the affects and effects of that something that was there even as the depths of time conceal its original purpose and identity.

Of use here might be the concept of *Nachleben* coined by Aby Warburg and applied to the study of afterlives by Marek Tamm (Tamm 2015:9–10; Tamm and Olivier 2019:5–6). *Nachleben* is not so much an “afterlife,” as the translation of the word implies, but more of a survival and continuation, a state where past, present, and future entangle with each other. Going back to the Latin *vestïgium*, a footstep, footprint, trace, or mark has no intrinsic historicity. While archaeologists can date footprints of ancient humans through stratigraphy and other indirect means, footprints themselves bear no date. They are haunting witnesses of that consequence that cuts through chronology. Thus, the issue with vestiges is that they are inherently unpredictable things that transgress all abilities and desires to control their uses, meanings, and chronologies. As long as a landscape vestige remains recognizable to itself as some spectral trace of human action that refuses to disappear under redevelopment or paused through museumification, it will continue to be a vestige cutting beyond its temporal and geographical boundaries.

This preoccupation with afterlives and vestiges is not merely a theoretical fixation. Rather they highlight the unacknowledged geographical extent and the limitations of current lifecycle planning within the resource extraction industry. They represent a material spill of extraction zones and material culture that stretches outward beyond the factory gates of Severonickel and the Wabush mines. Even the seemingly minor observations of industrial equipment being reused in and around Rizh-Guba and the Wabush sand pits should not be overlooked as they represent the steady diffusion of industrial objects and equipment outward into the surrounding spaces.

In a global context, the case studies presented here are just two examples of a larger assemblage that includes other quarries, forest cuts, runways, farms, communication towers, transportation networks, cable and electrical lines, and power stations that ripple outward in a network along the extractive frontiers around the world to feed distant industrial centers and the so-called postindustrial societies of many Western nations. Examples of similar phenomena have been documented elsewhere—in the North West Territories where Indigenous trappers use and maintain former cut lines for traditional trapping purposes (LeClerc and Keeling 2015), in Alberta where ATV users employ seismic lines from oil surveys for recreational use (Lee and Boutin 2006), and during my own time in Labrador where many people mentioned moose and wolves utilizing hundreds of kilometers of used and disused roads and electrical corridors as pathways deeper into the region, displacing the smaller caribou from their native habitat.

The concept of vestige thus allows scholars to talk more constructively about the landscape of ancillary impacts surrounding resource extraction operations. As implied by Olivier and other scholars (Olivier 2011; Hopper 2020), it could in fact be argued that all landscapes are made up of such vestiges, at least those that do not artificially seek to arrest decay in favor of museumification. While in Olivier’s discussions, vestiges allow a shift in our thinking from a past that is sequential to one that is about durations and memories—a position I generally support—I hesitate to jump on the claim that everything about Wabush and Monchegorsk is vestigial as it overlooks the newness, the speed, and the disregard of the lives and pasts of Indigenous people that defined their initial construction, elements that have come to, in part, characterize the last century (González-Ruibal 2008). For me in this article, vestige is a heuristic tool to understand the afterlives of the dispersed network of ancillary operations surrounding the Wabush mines and the Severonickel plant.

Similar work should be done on other ancillary operations that supported industrial, public, or even military developments in many other regions to trace networks of widespread unintended transformations that followed in their wake. While such ancillary operations would be present in all periods, their size and proliferation today are characteristically “supermodern” (Augé 1995; González-Ruibal 2008, 2019). As described in this article, the demands of garden city design in Wabush precipitated the extraction of sand, gravel, and topsoil in the sand pits. At the same time, while the extraction at Rizh-Guba began with hand tools not dissimilar to those used since the Iron Age, the demands of accelerating rates of production expanded the extraction at Rizh-Guba to mass destruction levels. Like the ruins of the south (González-Ruibal 2017), the scars left behind by the collective sum of ancillary operations create a mass of matter that is too big, too unwieldy, and often too overlooked to have anything done with them. It is excess matter, and from this excess that the quarries in my case studies have such diverse and multifaceted afterlives.

As such, my analysis here reveals that landscapes marked by abandoned ancillary operations are ones of unpredictability as human planning and intentionality are constantly surpassed. Disentangled from their industrial enterprises, they are released from being “things-for-us” (Pétursdóttir 2014:339) and cut outwards as marks and traces of those same industrial developments deeper and further in the pluritemporal *Nachleben*. Such work speaks to the broader discourse currently playing out regarding sustainability and remediation within the resource extraction industry (for some examples, see Sandlos and Keeling [2012, 2016], Didyk and Rjabova [2014], Storm [2014], Plisetskiy and Malitskaya [2017], Beckett and Keeling [2018], Nedoseka and Zhigunova [2019], and Tolvanen et al. [2019]). While making resource extraction more socially responsible and “green” is commendable in principle, the unpredictable vestigial qualities of ancillary impacts of resource extraction highlights the limits of human ability to account for the unforeseen material and temporal spill that extrudes from such activities.

## Conclusion

To contribute to the rich tradition of industrial and contemporary archaeology, I focused on exploring the unruly legacies of two ancillary operations of resource development in Russia and in Canada with the understanding that these are just a part of a large constellation of supporting features that blur the boundaries of resource extraction facilities. To facilitate their understanding, I relied on the term “vestige,” as has become popular for use among scholars. By grounding the definition in the work of Laurent Olivier (2011) and returning to the etymological roots of the word, I highlight how a vestige is that which maintains a part of its own substance and identity while being an agential cut across time and space.

Vestiges that represent disused ancillary impacts surrounding extractive industry development form active networks of fragmentating, evolving, and spatiotemporally uneven palimpsests. “Vestige” is a particularly useful concept in this context because it highlights the fact that abandonment and disuse is not an end in itself that leaves the trace ready for rehabilitation, revegetation, or museumification. Rather, a vestige is active and contains potential for reanimation and reactivation as human and nonhuman actors reengage with it in new and unforeseen ways. The ahistorical *vestïgium* of the resource extraction industry facilitates constellations of becomings in the long-lasting material consequence of the event that caused it.

Both at the quartzite quarry of Rizh-Guba and at the Wabush sand pits, I observed these various becomings through fragmentation, reinscription, and remobilization in and around the vestiges that facilitated new and unique uses that at times went beyond their industrial origins. While currently they provide spaces for rest, relaxation, creativity, and unstructured recreation, many of these reuses maintain the characteristics of these features by utilizing their material affordances in fluid and unpredictable ways.

In this context, the vestiges of the Rizh-Guba quarry and the Wabush sand pits continue to push the temporal and geographical limits of the resource extraction industry outward. This reveals an uncomfortable fact about resource extraction; when examined through the concept of vestige, the ancillary impacts of resource development expose how resource extraction escapes the geographical and temporal boundaries of the extraction and processing site and spreads outward as a pluritemporal collection of unruly conglomerations.

These observations are important to consider in our understanding of the dispersed impacts and legacies of resource extraction both in the present and the past. How different would the legacies of the industrial revolution look if scholars accounted for the entirety of constellations of ancillary industries that arose, declined, and evolved in parallel to and in the aftermath of the primary industries that they supported? How different would the legacies of the current industrial age appear? As illustrated by Lawrence, Davies, and Turnbull (2016, 2017), dispersed infrastructure networks carry a heavy anthropogenic effect. Even though, individually, ancillary impacts are small and benign compared to their core industrial operations, they are large in their total accumulated volume, geographical scope, and temporal durations. They are multiple and dispersed and often do not draw the same amount of attention and care that the core industrial operations do. Thus, it is inevitable for some of them to escape during industrial decline or restructuring to become vestiges on their own trajectories that carry the deep cuts of the undying legacies of extractive industry.

## Footnotes

1 The Iron Ore Company of Canada’s film “Ore in ’54,” made to promote and celebrate the company’s achievements, begins by calling the interior of the Labrador Peninsula “lonely, vast, and remote” and “neglected for centuries” (Les Studios Cinécraft 1954). While the interior of the Labrador Peninsula was neglected by Western business interests (aside from the fur trade taking place since the late 18th century), this conspicuously ignored the thousands of years of Indigenous history in the region (Loring et al. 2003; Neilsen 2016).

2 According to Statistics Canada Website (<https://www12.statcan.gc.ca>), the 2016 population of Labrador City was 7,220 and Wabush was 1,906.

3 An interesting additional perfective also comes in a footnote in Gordillo (2014:271), where *vestigios* is a word used by local people in northern Argentina to refer to piles of rubble without a defined form, which points to the vague, indeterminate state that vestiges can often occupy.

## References

[CR1] Allemann, Lukas 2013 *The Sami of the Kola Peninsula: About the Life of an Ethnic Minority in the Soviet Union*. Vol. 19, *Skriftserie*. Centre for Sami Studies, University of Tromsø, Tromsø, Norway.

[CR2] Andersson DT, Olsen B, Pétursdóttir Þ (2014). No Man's Land: The Ontology of a Space Left Over. *Ruin Memories: Materiality, Aesthetics and the Archaeology of the Recent Past*.

[CR3] Arsenault W (1997). Ashuanipi-Duley Airlift. Them Days.

[CR4] Augé M (1995). *Non-Places: An Introduction to Supermodernity*.

[CR5] Baeten J, Langston N, Lafreniere D (2018). A Spatial Evaluation of Historic Iron Mining Impacts on Current Impaired Waters in Lake Superior's Mesabi Range. Ambio.

[CR6] Beckett C, Keeling A (2018). Rethinking Remediation: Mine Reclamation, Environmental Justice, and Relations of Care. Local Environment.

[CR7] Beljunas 1938 *Объяснительная Записка по вопросу образования Риж-Губского сельсовета Мончегорского р-на* (Explanatory note on the question of development of Rizh-Guba soviet in Monchegorsk region). Председатель Мончегорского Горплана, Monchegorsk, Russia.

[CR8] Bogomolov, V. 1957 Красивая Тундра (Beautiful tundra). In *У Карты Кольского Полуострова: Сборник Очерков о Прошлом и Настоящем Мурманской Области*, pp. 76–79. Книжная Редакция Полярной Правды, Murmansk, Russia.

[CR9] Bolotova A (2012). Loving and Conquering Nature: Shifting Perceptions of the Environment in the Industrialised Russian North. Europe-Asia Studies.

[CR10] Bolotova, Alla 2014 Если Ты Полюбишь Север, Не Разлюбишь Никогда: Взаимодействие с Природой в Северных Промышленных Городах (If you fall in love with the North, you will never stop: Concern with nature in northern industrial towns). *Неприкосновенный Запас* 97(5).

[CR11] Boutet, Jean-Sébastien 2012 An Innu-Naskapi Ethnohistorical Geography of Industrial Iron Mining Development at Schefferville, Québec. Master's thesis, Department of Geography, Memorial University of Newfoundland, St. John’s, NL.

[CR12] Boutet J-S (2013). Opening Ungava to Industry: A Decentring Approach to Indigenous History in Subarctic Québec, 1937–54. Cultural Geographies.

[CR13] Bradbury JH (1983). Declining Single-Industry Communities in Quebec-Labrador, 1979–1983. Journal of Canadian Studies.

[CR14] Bradbury JH (1984). The Impact of Industrial Cycles in the Mining Sector: The Case of the Quebec-Labrador Region in Canada. International Journal of Urban and Regional Research.

[CR15] Bradbury JH (1985). The Rise and Fall of the “Fourth Empire of the St. Lawrence”: The Québec-Labrador Iron Ore Mining Region. Cahiers de géographie du Québec.

[CR16] Britskaya, Tatyana 2019 Inhale this Holiday Greeting. *Novaya Gazeta* 15 March.

[CR17] Bruno A (2016). *The Nature of Soviet Power*.

[CR18] Buchli V, Lucas G (2001). *Archaeologies of the Contemporary Past*.

[CR19] Casella EC, Symonds J (2005). *Industrial Archaeology: Future Directions*.

[CR20] Dezhkina, G. N. 2015 *Мы Жили По-Соседству: История Рабочих Поселков Мончегорска* (We lived as neighbors: History of working villages of Monchegorsk). Муниципальное бюджетное учреждение культуры, “Мончегорская централизованная библиотечная система,” центральная городская библиотека, Monchegorsk, Russia.

[CR21] Didyk, Vladimir Vsevolodovich, and Larisa Aleksandrovna Rjabova 2014 Моногорода российской Арктики: стратегии развития (на примере Мурманской области) (Monotowns of the Russian Arctic: Strategic development (with the example of Murmansk region). *Экономические и Социальные Перемены: Факты, Тенденции, Прогноз* 4(34):84–99.

[CR22] Domanska E (2017). *Necros: An Ontology of Human Remains*, Eliza Rose and Paul Vickers, translators.

[CR23] D. W. Knight Associates (1985). *The Town of Wabush*.

[CR24] Edensor T (2005). The Ghosts of Industrial Ruins: Ordering and Disordering Memory in Excessive Space. Environment and Planning D: Society and Space.

[CR25] Edensor T (2005). Waste Matter: The Debris of Industrial Ruins and the Disordering of the Material World. Journal of Material Culture.

[CR26] Farstadvoll S (2019). Growing Concerns: Plants and Their Roots in the Past. Journal of Contemporary Archaeology.

[CR27] Farstadvoll, Stein 2019b A Speculative Archaeology of Excess: Exploring the Afterlife of a Derelict Landscape Garden. Doctoral dissertation, Department of Archaeology, History, Religious Studies and Theology, UiT: The Arctic University of Norway, Tromsø, Norway.

[CR28] Farstadvoll S (2019). Vestigial Matters: Contemporary Archaeology and Hyperart. Norwegian Archaeological Review.

[CR29] Genge, Ngaire 2009 Unseasonable Weather Causes Extreme Dust Lift Off over Towns. *53 North*, 17 May, 6(12):5.

[CR30] Geren R, McCullough B (1990). *Cain’s Legacy: The Building of Iron Ore Company of Canada*.

[CR31] González-Ruibal A (2008). Time to Destroy: An Archaeology of Supermodernity. Current Anthropology.

[CR32] González-Ruibal A, McAtackney L, Ryzewski K (2017). Ruins of the South. *Contemporary Archaeology and the City: Creativity, Ruination, and Political Action*.

[CR33] González-Ruibal A (2019). *An Archaeology of the Contemporary Era*.

[CR34] Goodyear S (2012). Cocaine Use in Labrador West Grows along with the Economy. Labrador Life.

[CR35] Goodyear S (2015). Wabush still Suffering after Mine Shut Down. Labrador Life.

[CR36] Gordillo GR (2014). *Rubble: The Afterlife of Destruction*.

[CR37] Gutsol N, Vinogradova S, Samorukova A (2007). *Kola Saami Relocated Groups*.

[CR38] Harrison M (2003). The “Impossible” Railroad. Them Days.

[CR39] Harrison R (2011). Surface Assemblages. Towards an Archaeology in and of the Present. Archaeological Dialogues.

[CR40] Harrison R, Harrison R, DeSilvey C, Holtorf C, Macdonald S, Bartolini N, Breithoff E, Fredheim H, Lyons A, May S, Morgan J, Penrose S (2020). Heritage as Future-Making Practices. *Heritage Futures: Comparative Approaches to Natural and Cultural Practices*.

[CR41] Harrison R, Schofield J (2010). *After Modernity: Archaeological Approaches to the Contemporary Past*.

[CR42] Hilton, Keith David 1968 The Iron Mining Communities of Quebec-Labrador: A Study of a Resource Frontier. Master’s thesis, Department of Geography, McGill University, Montreal, QC.

[CR43] Hopper, Justin 2020 Uncanny Landscapes #4—Stein Farstadvoll, 13 August [podcast]. Uncanny Landscapes, PodBean <https://uncannylandscapes.podbean.com/>. Accessed 16 February 2023.

[CR44] Hynes D (1990). Town of Wabush History.

[CR45] Hynes T (2021). *Workhorse*.

[CR46] Institut “Giprogor” [2010] *Карта-Схема Зонирования Территорий Границ г. Мончегорска с Подведомственной Территорией* (Map-plan for zoning of the territorial border of the town of Monchegorsk with the subordinate territory). Открытое Акционерное Общество Российский Институт Градостроительства и Инвестиционного Развития, Moscow, Russia. Institut “Giprogor” [2010] Карта-Схема Зонирования Территорий Границ г. Мончегорска с Подведомственной Территорией (Map-Plan for Zoning of the Territorial Border of the Town of Monchegorsk with the Subordinate Territory). Открытое Акционерное Общество Российский Институт Градостроительства и Инвестиционного Развития, Moscow, Russia.

[CR47] Jones A (2007). *Memory and Material Culture*.

[CR48] Keeling A (2010). “Born in an Atomic Test Tube”: Landscapes of Cyclonic Development at Uranium City, Saskatchewan. The Canadian Geographer/Le Géographe canadien.

[CR49] Keeling A, Sandlos J, Bocking S, Martin B (2017). Ghost Towns and Zombie Mines: The Historical Dimensions of Mine Abandonment, Reclamation, and Redevelopment in the Canadian North. *Ice Blink*.

[CR50] Kraevedcheskij Portal Monchegorska 2017 Симбиоз Эльвириной Дачи. (Symbiosis of Elvira’s dacha). Краеведческий Портал Мончегорска <http://krai.monlib.ru/simbioz-elvirinoj-dachi-elvira-nikolaevna-kozhevnikova/>. Accessed 26 February 2023.

[CR51] Lawrence S, Davies P, Turnbull J (2016). The Archaeology of Anthropocene Rivers: Water Management and Landscape Change in “Gold Rush” Australia. Antiquity.

[CR52] Lawrence S, Davies P, Turnbull J (2017). The Archaeology of Water on the Victorian Goldfields. International Journal of Historical Archaeology.

[CR53] LeCain TJ (2009). *Mass Destruction: The Men and Giant Mines that Wired America and Scared the Planet*.

[CR54] LeCain TJ, Olsen B, Pétursdóttir Þ (2014). The Ontology of Absence: Uniting Materialist and Ecological Interpretations at an Abandoned Open-pit Copper Mine. *Ruin Memories: Materiality, Aesthetics and the Archaeology of the Recent Past*.

[CR55] LeClerc E, Keeling A (2015). From Cutlines to Traplines: Post-Industrial Land Use at the Pine Point Mine. Extractive Industries and Society.

[CR56] Lee P, Boutin S (2006). Persistence and Developmental Transition of Wide Seismic Lines in the Western Boreal Plains of Canada. Journal of Environmental Management.

[CR57] Les Studios Cinécraft (1954). *Ore in '54*.

[CR58] Loring S, McCaffrey MT, Armitage P, Ashini D (2003). The Archaeology and Ethnohistory of a Drowned Land: Innu Nation Research along the Former Michikamats Lake Shore in Nitassinan (Interior Labrador). Archaeology of Eastern North America.

[CR59] Lucas G (2015). Archaeology and Contemporaneity. Archaeological Dialogues.

[CR60] Lukichev, J. S. 1993 *Город в Красивой Тундре* (Town in the beautiful tundra). Мурманское Издательско-Полиграфическое Предприятие “Север,” Murmansk, Russia.

[CR61] Maher, Patrick 1992 Labrador City’s Past: An Interview with Mr. Patrick Maher on the Iron Ore Company of Canada, Jacqueline Gallant, editor. Manuscript, Labrador City Public Library, Labrador City, NL.

[CR62] Marcil, Dorice, and Agnes Greene 1992 Labrador City’s Past: Going Back in Time with Dorice Marcil and Agnes Greene, Jacqueline Gallant, editor. Manuscript, Labrador City Public Library, Labrador City, QC.

[CR63] Martin P, Douet J (2012). Industrial Archaeology. *Industrial Heritage Retooled: The TICCIH Guide to Industrial Heritage Conservation*.

[CR64] McLean R (1995). A Trip on the Trapline. Them Days.

[CR65] McQuire S (2019). One Map to Rule Them All? Google Maps as Digital Technical Object. Communication and the Public.

[CR66] Moiseenko TI, Morgunov BA, Gashkina NA, Megorskiy VV, Pesiakova AA (2018). Ecosystem and Human Health Assessment in Relation to Aquatic Environment Pollution by Heavy Metals: Case Study of the Murmansk Region, Northwest of the Kola Peninsula, Russia. Environmental Research Letters.

[CR67] *Monchegorskij Rabotnik* 1954 День Поселка Риж-Губа (A day in the village Rizh-Guba). *Мончегорский Работник* 25 July:3.

[CR68] Nedoseka EV, Zhigunova GV (2019). Features of Local Identity of Single-Industry Town Residents (The case of the Murmansk Oblast). Arctic and North.

[CR69] Neilsen, Scott 2016 An Archaeological History of Ashuanipi, Labrador. Doctoral dissertation, Department of Archaeology, Memorial University of Newfoundland, St. John’s, NL.

[CR70] Nilsen, Thomas 2019 Old Copper Smelter in Monchegorsk Faces Closure, Brand New Under Consideration. *Barents Observer* 21 November,

[CR71] Nilsen, Thomas 2021 Old Copper Plant Closed for Good. *Barents Observer* 2 March.

[CR72] Olivier, Laurent 2011 *The Dark Abyss of Time: Archaeology and Memory*, Arthur Greenspan, translator. AltaMira Press, Lanham, MD.

[CR73] Olsen B, Graves-Brown P, Harrison R, Piccini A (2013). Memory. *The Oxford Handbook of the Archaeology of the Contemporary World*.

[CR74] Olsen B, Pétursdóttir Þ (2016). Unruly Heritage Tracing Legacies in the Anthropocene. Arkæologisk Forum.

[CR75] Olsen B, Vinogradova S (2019). (In)significantly Soviet: The Heritage of Teriberka. International Journal of Heritage Studies.

[CR76] *Oxford English Dictionary* 2020 Vestige, n. In *Oxford English Dictionary*. Oxford University Press, Oxford, UK.

[CR77] Palmer M, Neaverson P (1998). *Industrial Archaeology: Principles and Practice*.

[CR78] Palmer M, Orange H (2016). The Archaeology of Industry: People and Places. Post-Medieval Archaeology.

[CR79] Parlee BL, Sandlos J, Natcher DC (2018). Undermining Subsistence: Barrel-Ground Caribou in a “Tragedy of Open Access”. Science Advances.

[CR80] Pétursdóttir, Þóra 2013 Concrete Matters towards an Archaeology of Things. Doctoral dissertation, Department of Archaeology and Social Anthropology, University of Tromsø, Tromsø, Norway.

[CR81] Pétursdóttir Þ, Olsen B, Pétursdóttir Þ (2014). Things Out-of-Hand: The Aesthetics of Abandonment. *Ruin Memories: Materiality, Aesthetics and the Archaeology of the Recent Past*.

[CR82] Piper L (2009). *The Industrial Transformation of Subarctic Canada*.

[CR83] Plisetskiy EE, Malitskaya EA (2017). The Features of State and Municipal Management of the Development of Single-Industry Settlements in the Arctic Zone of the Russian Federation. Arctic and North.

[CR84] Ponte A, Kowal S, Hutton J (2008). “Making the North”: Mines and Towns of the Labrador Trough. *Landscript 05*.

[CR85] Poznjakov, V. J. 1999 *Североникель* (Severonickel). ГУП Издательский дом “Руда и Металлы,” Moscow, Russia.

[CR86] Riggs T (2019). Life was Easy in Wabush. Them Days.

[CR87] Rompkey B (2003). *The Story of Labrador*.

[CR88] ROSSTAT, Federal′naja Sluzhba Gosudarstvennoj Statistiki 2018 Численность Постоянного Населения Российской Федерации по Муниципальным Образованиям на 1 Января 2018 Года (Number of full-time residents of the Russian Federation on the Municipal Level on 1 January 2018). Федеральная Служба Государственной Статистики, Moscow, Russia.

[CR89] Sandlos, John, and Arn Keeling 2012 Claiming the New North: Development and Colonialism at the Pine Point Mine, Northwest Territories, Canada. *Environment and History* 18(1):5–34.

[CR90] Sandlos J, Keeling A (2016). Toxic Legacies, Slow Violence, and Environmental Injustice at Giant Mine, Northwest Territories. Northern Review.

[CR91] Schoenauer N (1976). Fermont: A New Version of the Company Town. JAE.

[CR92] Shanks M (2012). *The Archaeological Imagination*.

[CR93] Stantec (2019). Land Use Zoning Map.

[CR94] Stewart H, Jungkind K, Losey R (2020). Life on the Fence Line. Early 20th-century Life in Ross Acreage. Archaeological Dialogues.

[CR95] Storm A (2014). *Post-Industrial Landscapes Scars*.

[CR96] Tamm M, Tamm M (2015). Introduction: Afterlife of Events: Perspectives on Mnemohistory. *Afterlife of Events: Perspectives of Mnemohistory*.

[CR97] Tamm M, Olivier L, Tamm M, Olivier L (2019). Introduction: Rethinking Historical Time. *Rethinking Historical Time: New Approaches to Presentism*.

[CR98] Thistle J, Langston N (2016). Entangled Histories: Iron Ore Mining in Canada and the United States. Extractive Industries and Society.

[CR99] Tolvanen A, Eilu P, Juutinen A, Kangas K, Kivinen M, Markovaara-Koivisto M, Naskali A, Salokannel V, Tuulentie S, Simila J (2019). Mining in the Arctic Environment: A Review from Ecological, Socioeconomic and Legal Perspectives. Journal of Environmental Management.

[CR100] Venovcevs, Anatolijs 2020 Twin Falls: Labrador's Unruly Industrial Heritage. In *Provincial Archaeology Office Annual Review 2019*, Stephen Hull and Martha Drake, editors, pp. 214–226. Department of Tourism, Culture, Industry and Innovation, St. John’s, NL.

[CR101] Venovcevs, Anatolijs 2021 Living with Socialism: Toward an Archaeology of a Post-Soviet Industrial Town. *Extractive Industries and Society* 8(4).

[CR102] Wheelersburg RP, Gutsol N (2008). Babinski and Ekostrovski: Saami Pogosty on the Western Kola Peninsula, Russia from 1880 to 1940. Arctic Anthropology.

[CR103] Young, Ewart [1964] *Souvenir Booklet of the Wabush-Labrador City Area*. Northern News and Varieties Limited, St. John’s, NL.

[CR104] Zaslow M (1988). *The Northward Expansion of Canada, 1914*–*1967*.

